# Association between ethnicity and under-5 mortality: analysis of data from demographic surveys from 36 low-income and middle-income countries

**DOI:** 10.1016/S2214-109X(20)30025-5

**Published:** 2020-02-19

**Authors:** Cesar G Victora, Aluisio J D Barros, Cauane Blumenberg, Janaina Calu Costa, Luis Paulo Vidaletti, Fernando C Wehrmeister, Bruno Masquelier, Lucia Hug, Danzhen You

**Affiliations:** a International Center for Equity in Health, Federal University of Pelotas, Pelotas, Brazil; b Centre de Recherche en Démographie, Université Catholique de Louvain, Louvain, Belgium; c Division of Data, Analytics, Planning and Monitoring, UNICEF, New York, NY, USA

## Abstract

**Background:**

The UN Sustainable Development Goals (SDGs) call for stratification of social indicators by ethnic groups; however, no recent multicountry analyses on ethnicity and child survival have been done in low-income and middle-income countries (LMICs).

**Methods:**

We used data from Demographic and Health Surveys and Multiple Indicator Cluster Surveys collected between 2010 and 2016, from LMICs that provided birth histories and information on ethnicity or a proxy variable. We calculated neonatal (age 0–27 days), post-neonatal (age 28–364 days), child (age 1–4 years), and under-5 mortality rates (U5MRs) for each ethnic group within each country. We assessed differences in mortality between ethnic groups using a likelihood ratio test, Theil's index, and between-group variance. We used multivariable analyses of U5MR by ethnicity to adjust for household wealth, maternal education, and urban–rural residence.

**Findings:**

We included data from 36 LMICs, which included 2 812 381 livebirths among 415 ethnic groups. In 25 countries, significant differences in U5MR by ethnic group were identified (all p<0·05 likelihood ratio test). In these countries, the median mortality ratio between the ethnic groups with the highest and lowest U5MRs was 3·3 (IQR 2·1–5·2; range 1·5–8·5), whereas among the remaining 11 countries, the median U5MR ratio was 1·9 (IQR 1·7–2·5; range 1·4–10·0). Ethnic gaps were wider for child mortality than for neonatal or post-neonatal mortality. In nearly all countries, adjustment for wealth, education, and place of residence did not affect ethnic gaps in mortality, with the exception of Guatemala, India, Laos, and Nigeria. The largest ethnic group did not have the lowest U5MR in any of the countries studied.

**Interpretation:**

Significant ethnic disparities in child survival were identified in more than two-thirds of the countries studied. Regular analyses of ethnic disparities are essential for monitoring trends, targeting, and assessing the impact of health interventions. Such analyses will contribute to the effort towards leaving no one behind, which is at the centre of the SDGs.

**Funding:**

Bill & Melinda Gates Foundation, UNICEF, Wellcome Trust, Associação Brasileira de Saúde Coletiva.

## Introduction

The 2030 Agenda for Sustainable Development, adopted by all UN member states in 2015, provides a shared blueprint for peace and prosperity for people and the planet, and is committed to ensuring that no one is left behind. Regarding child survival specifically, the UN Sustainable Development Goal (SDG) 3.2 aims to “end preventable deaths of newborns and children under 5 years of age”. To achieve this goal, countries should strive to ensure that life-saving interventions are accessible for all children, with a specific focus on subgroups at high risk of mortality. To identify these children, SDG 17.18 calls for countries to “increase significantly the availability of high-quality, timely and reliable data disaggregated by income, gender, age, race, ethnicity, migratory status, disability, geographic location and other characteristics relevant in national contexts”.

Analyses of intracounty inequalities in child survival stratified by wealth, maternal education, sex, and geographical location are widely available in peer-reviewed literature from low-income and middle-income countries (LMICs), and in reports and websites produced by international organisations.^1^, ^2^, ^3^ However, few systematic, multicountry analyses of ethnic inequalities in child health and survival in LMICs have been done. Ethnicity is a complex construct comprising culture, diet, language, and ancestry that is associated with variations in health beliefs and behaviours.^4^ Ethnicity is also a key factor in social cohesion, and thus in the dissemination of health information. As a framework of identification, sometimes reactivated for political reasons, ethnicity can be associated with unequal access to socioeconomic opportunities and public goods between different sectors of the population.

Most existing analyses on ethnic inequalities in LMICs are limited to comparisons between indigenous and non-indigenous subpopulations within one or more countries.^3^, ^5^, ^6^, ^7^, ^8^, ^9^ We found only one multicountry publication on under-5 mortality rate (U5MR) for multiple ethnic groups done in 11 countries in sub-Saharan Africa.^10^ In addition to being limited to African countries, that study only compared one or two ethnicities in each country with the rest of the national population.

**Research in context**

**Evidence before this study**

We searched PubMed from inception to Aug 31, 2019, using the search terms “developing countries” OR “low-and-middle-income)” AND (“infant mortality”[Mesh] OR “underfive mortality” OR “under five mortality” OR “under-five mortality”) AND (ethnicity OR race)”, without any language restrictions. Our search identified a single article published in 2000, which included multicountry analyses of child mortality by ethnicity. The authors compared child mortality among one or two privileged ethnic groups (ie, groups assumed to have lower child mortality because of urban residence, wealth, education, nutritional status, and access to services) with the rest of the population in 11 sub-Saharan African countries. In ten of these countries, child mortality was significantly lower among the privileged ethnic groups than all other ethnicities combined. Multivariable analyses showed that sociodemographic variables and health-care utilisation accounted for a substantial fraction of the disparities observed between different ethnic groups. Our search also identified several analyses done in single low-income and middle-income countries (LMICs) that showed ethnic differences in mortality.

**Added value of this study**

Our analyses of 415 ethnic groups in 36 countries were based on all standardised demographic and health surveys done since 2010. In 25 countries, significant differences in under-5 mortality rate (U5MR) were identified between ethnic groups. In these countries, the median mortality ratio between the ethnic groups with the highest and lowest mortality rates was 3·3. The largest ethnic groups did not have the lowest U5MR in any of the countries studied. Adjustment for family wealth, maternal education, and urban–rural residence reduced, but did not eliminate the differences in mortality identified between ethnic groups in most countries. Our analyses provide a better understanding of the inequalities in mortality associated with ethnicity and identified substantial disparities across ethnic groups in most of the countries studied.

**Implications of all the available evidence**

Despite the recommendation in the UN Sustainable Development Goal (SDG) 17.18 for disaggregation of indicators according to ethnicity, literature on ethnic inequalities in child survival is scarce. We provide the most comprehensive analyses on this topic to date, revealing wide gaps in most countries studied. Regular analyses of ethnic disparities in U5MR in LMICs are essential for monitoring trends, and for targeting and assessing the impact of health interventions. Such analyses will contribute to the efforts towards leaving no one behind, which is at the centre of the SDGs.

In this study, we aimed to assess whether significant differences exist in U5MR between ethnic groups within countries, using data from nationally representative household surveys done in 36 LMICs, and we aimed to estimate the magnitude of such differences, and to assess whether the differences persisted after adjustment for household wealth, maternal education, and place of residence.

## Methods

### Data sources and procedures

We used publicly available data from household sample surveys done in 98 LMICs between 2010 and 2016, and selected data from 36 LMICs with available information on birth histories and either ethnicity or a proxy variable, such as language spoken at home. For 28 countries data were extracted from Demographic and Health Surveys (DHS) and for eight countries data were extracted from Multiple Indicator Cluster Surveys (MICS). The two survey programmes are highly comparable with regard to sampling and questionnaires used.^11^, ^12^ We used broad definitions of ethnicity, including self-reported ethnic affiliation, language, skin colour (in South Africa), and caste or tribal group (in India). For countries with more than one survey, we selected the most recent.

Within each sampled household, women aged 15–49 years are eligible to participate, and those who gave consent provided information on their birth histories and on characteristics of the household. Two questionnaires were used: data on ethnicity or language were collected for women aged 15–49 years in DHS, and for the head of the household in MICS. In eight countries, the information was about the language spoken at home, in South Africa the information was about skin colour, in India the information was about caste or tribe, and for the remaining countries the information referred to ethnicity or tribe. The ethnic groups in each country are listed in the appendix (pp 2–13). Herein, we use the term ethnicity to indicate ethnic group, language, skin colour, or caste.

Ethics approval was obtained by the institutions that administered the surveys and all analyses used anonymised databases.

### Statistical analysis

U5MRs and 95% CIs were calculated using survival analyses based on deaths that occurred in the 10 years before the surveys were done, which is the standard approach for stratified analyses (eg, for wealth quintiles, maternal education).^13^ These methods are described in the appendix (pp 14–15). Using the same procedure, we also estimated neonatal (age 0–27 days), post-neonatal (age 28–364 days), and child (age 1–4 years) mortality rates by ethnicity.

To assess the ethnic differences in U5MR within each country, we used a likelihood ratio test to compare a model including age of the child with another model including age and ethnic group. We also calculated two summary indices for quantifying relative and absolute inequalities in categorical variables.^14^ The magnitude of relative inequalities, expressed as ratios among ethnic groups, was estimated using Theil's index,^14^ which takes into account the proportion of the population in each group and the mortality ratios in each ethnicity relative to the national mean value. The index equals a value of zero when no inequality exists; as relative inequality increases, the value increases, with no upper bound. The index is most influenced by large ethnic groups with mortality rates that are substantially different from the national rate. In our analyses the original index values were multiplied by 1000 to facilitate interpretation. Further details on the index, formula, and interpretation are provided in the appendix (pp 16–19). Absolute inequalities, expressed as differences among ethnic groups, were calculated with a between-group variance indicator. Further details are also shown in the appendix (pp 16–19). SEs for both indices were estimated by resampling the observations of each ethnicity per country 50 times (with replacement). Negative 95% CI values were truncated at zero. We have focused on relative inequalities since statistical comparisons of mortality rates are usually expressed as ratios.

We calculated the U5MR ratio and the corresponding 95% CI between the ethnic groups with the highest and lowest mortality rates in each country. We have presented median U5MR ratios with corresponding IQRs and ranges. We used a non-parametric medians test to compare the magnitude of Theil's index and between-group variance for neonatal, post-neonatal, and child mortality by ethnicity.

Three covariates were used in multivariable analyses. Maternal education was categorised in three groups on the basis of self-report: none (no formal education); primary (any primary education, including completed primary education); and secondary or higher (any secondary education, including complete secondary). Urban or rural residence was categorised according to country-specific delimitations at the time of the survey. Household wealth indices included in the DHS and MICS datasets were used in the analyses. These indices were derived using principal component analyses of household assets and characteristics of the building, presence of electricity, water supply and sanitary facilities, in addition to other variables associated with wealth. Since relevant assets might vary between urban and rural households, separate principal component analyses were done in each area, which were later combined into a single score using a scaling procedure to allow comparability between urban and rural households.^15^

Data analyses were done using Stata (version 15.0) for descriptive analyses. Estimation of mortality was done using the Stata module syncmrates and the R statistical software (version 3.6.1) was used for adjustment for covariates in a Poisson regression framework (household wealth, women's education, and urban–rural residence). For Poisson regression, the reference category was the ethnic group with the largest number of livebirths in the survey sample. Adjusted mortality rates were obtained by multiplying the rate ratios by the mortality rate in the reference category. For the adjusted mortality rates, we assumed that ethnic differentials were constant by age in children aged younger than 5 years.

All analyses accounted for the complex survey design. Mortality estimates for which the coefficient of variation was greater than 15% were flagged to indicate lower precision, consistent with cutoffs of 10–20% that have been used previously in the literature.^16^ Mortality estimates for ethnic groups with fewer than 200 births in the 10 years before the survey were omitted due to the high coefficients of variability observed for these groups.

### Role of the funding source

The funders did not have any role in study design, data analysis, data interpretation, writing of the report, or submission for publication. The corresponding author had full access to all the data in the study and had final responsibility for the decision to submit for publication.

## Results

After the exclusion of mortality estimates for ethnic groups with fewer than 200 births reported in the 10 years before the survey, we included survey data from 36 LMICs, which included 2 812 381 livebirths among 415 ethnic groups. The number of ethnic groups per country ranged from three in Guatemala to 32 in Zambia (table).

Of the 36 countries included, no differences in U5MR between ethnic groups were identified in 11 countries (all p>0·05 likelihood ratio test; table). Among these 11 countries, Theil's index for relative inequality was non-significant in six countries (Benin, The Gambia, Guyana, Honduras, Senegal, and South Africa) and significant in the other five countries (Republic of the Congo, Ethiopia, Gabon, Liberia, and Malawi); p values for the likelihood ratio test for these five countries ranged from 0·057 to 0·192. In Guatemala, the p value for the likelihood ratio test was 0·040; however, the Theil's index value was not significant. The between-group-variance indicator for absolute inequality showed similar results in terms of statistical significance to those obtained with Theil's index (appendix pp 16–19), although the rankings of countries differed in terms of relative and absolute inequalities. Overall, the three tests provided consistent results in terms of significance.

**Table:** U5MRs of the 36 countries included in the analyses

	**Year**	**Survey**	**Variable**	**Total number of births, n**	**Ethnic groups, n**	**U5MR by ethnic group (per 1000 livebirths)**			**Likelihood ratio p value**	Mortality ratio*(95% CI)	**Theil's index (95% CI)**
						Median (IQR)	Lowest (95% CI)	Highest (95% CI)			
Afghanistan	2015	DHS	Ethnicity	125 488	9	64 (56–73)	45 (23–67)	162 (138–185)	0·009	3·6 (2·4–4·8)	39·9 (30·9–48·9)
Angola	2015	DHS	Language	41 999	12	70 (50–101)	23 (0–53)	132 (87–176)	<0·0001	5·8 (3·2–8·3)	40·1 (28·1–52·0)
Benin	2014	MICS	Ethnicity	45 183	10	113 (99–117)	86 (52–120)	123 (103–144)	0·714	1·4 (1·0–1·8)	2·4 (0·0–5·2)
Burkina Faso	2010	DHS	Ethnicity	55 853	12	163 (125–185)	73 (55–92)	204 (166–241)	<0·0001	2·8 (2·0–3·5)	19·7 (13·6–25·8)
Cameroon	2014	MICS	Ethnicity	26 040	11	125 (81– 150)	58 (43–74)	206 (116–295)	<0·0001	3·5 (2·5–4·6)	68·0 (50·7–85·4)
Chad	2014	DHS	Ethnicity	67 356	22	139 (105–172)	38 (20–57)	215 (157–273)	<0·0001	5·6 (3·7–7·5)	50·4 (43·1–57·7)
Democratic Republic of the Congo	2013	DHS	Ethnicity	59 074	9	102 (100–119)	36 (0–76)	126 (102–151)	0·033	3·5 (2·2–4·8)	5·6 (2·5–8·7)
Republic of the Congo	2011	DHS	Ethnicity	31 688	11	95 (75–104)	53 (33–73)	125 (81–169)	0·192	2·4 (1·6–3·1)	10·0 (2·8–17·2)
Côte d'Ivoire	2011	DHS	Ethnicity	26 317	23	121 (102–146)	45 (3–87)	237 (177–296)	0·001	5·2 (3·6–6·9)	8·3 (26·1–57·0)
Ethiopia	2016	DHS	Ethnicity	39 494	19	88 (67–101)	13 (0–35)	127 (0–288)	0·095	10·0 (4·2–15·8)	41·6 (15·1–38·8)
Gabon	2012	DHS	Ethnicity	20 619	9	60 (57–72)	18 (0–41)	76 (54–98)	0·070	4·2 (2·0–6·3)	26·9 (4·9–33·8)
The Gambia	2013	DHS	Ethnicity	26 179	10	53 (49–65)	36 (2–70)	70 (50–89)	0·514	1·9 (1·2–2·7)	19·3 (0·0–16·6)
Ghana	2014	DHS	Ethnicity	23 117	9	62 (60–88)	59 (18–101)	102 (78–126)	0·009	1·7 (1·2–2·3)	7·1 (10·1–39·4)
Guatemala	2014	DHS	Ethnicity	55 300	3	42 (35–61)	34 (30–39)	62 (22–101)	0·040	1·8 (1·0–2·5)	24·8 (0·0–14·5)
Guinea	2012	DHS	Ethnicity	27 683	7	123 (89–148)	59 (0–136)	160 (141–180)	0·0002	2·7 (1·9–3·5)	7·2 (7·9–21·0)
Guinea-Bissau	2014	MICS	Language	27 477	7	88 (77– 126)	68 (55–80)	136 (120–151)	<0·0001	2·0 (1·4–2·6)	14·5 (23·7–49·4)
Guyana	2014	MICS	Ethnicity	11 122	4	33 (31–39)	29 (10–48)	44 (29–60)	0·400	1·5 (0·8–2·2)	36·5 (0·0–36·1)
Honduras	2011	DHS	Ethnicity	48 893	7	32 (28–49)	24 (6–42)	54 (38–70)	0·405	2·2 (1·2–3·3)	13·8 (0·0–26·5)
India	2015	DHS	Ethnicity	1 265 049	5	58 (53–59)	41 (39–43)	61 (58–63)	<0·0001	1·5 (0·9–2·1)	12·5 (7·1–9·6)
Kenya	2014	DHS	Ethnicity	83 571	23	55 (36–65)	18 (0–49)	95 (84–106)	<0·0001	5·2 (2·6–7·8)	59·3 (45·2–73·4)
Laos	2011	MICS	Ethnicity	56 710	4	98 (82–126)	80 (72–89)	138 (124–151)	<0·0001	1·7 (1·2–2·2)	30·3 (22·9–37·8)
Liberia	2013	DHS	Language	30 713	18	124 (89–142)	59 (33–85)	161 (73–250)	0·174	2·7 (1·9–3·5)	15·2 (8·3–22·0)
Malawi	2015	DHS	Ethnicity	68 074	11	70 (64–79)	58 (47–70)	104 (62–147)	0·057	1·8 (1·2–2·4)	7·0 (3·2–10·8)
Mali	2015	MICS	Ethnicity	55 772	11	117 (90–126)	36 (14–57)	157 (112–201)	<0·0001	4·4 (2·8–6·0)	31·0 (24·4–37·5)
Mozambique	2011	DHS	Ethnicity	37 877	19	106 (83–130)	55 (24–86)	161 (132–190)	<0·0001	2·9 (2·0–3·8)	26·8 (17·7–35·8)
Niger	2012	DHS	Language	43 831	6	106 (69–150)	65 (22–107)	164 (148–179)	<0·0001	2·5 (1·8–3·3)	16·0 (11·2–20·8)
Nigeria	2016	MICS	Ethnicity	101 691	4	82 (70–117)	67 (58–77)	144 (134–153)	<0·0001	2·1 (1·5–2·8)	41·8 (33·5–50·0)
Pakistan	2012	DHS	Language	49 913	15	94 (75–120)	30 (0–60)	185 (30–340)	<0·0001	6·1 (3·8–8·5)	31·7 (21·0–42·3)
Paraguay	2016	MICS	Language	14 355	5	16 (13–24)	8 (2–15)	71 (48–94)	0·002	8·5 (2·4–14·5)	174·5 (94·4–254·5)
Philippines	2013	DHS	Language	31 668	10	31 (27–45)	13 (0–27)	84 (38–129)	<0·0001	6·6 (2·7–10·4)	61·5 (37·5–85·6)
Senegal	2016	DHS	Ethnicity	22 546	7	64 (56–71)	48 (37–58)	71 (59–83)	0·170	1·5 (0·9–2·0)	13·6 (0·0–27·5)
Sierra Leone	2013	DHS	Ethnicity	46 941	10	162 (142–179)	113 (75–151)	198 (172–225)	0·0003	1·8 (1·3–2·2)	7·1 (4·2–10·0)
South Africa	2016	DHS	Ethnicity	14 031	3	40 (29–52)	29 (0–58)	52 (44–61)	0·529	1·8 (1·0–2·7)	5·5 (0·0–20·5)
Togo	2013	DHS	Ethnicity	26 152	7	102 (76–110)	46 (26–65)	121 (80–162)	<0·0001	2·6 (1·7–3·5)	19·1 (10·0–28·2)
Uganda	2016	DHS	Ethnicity	56 618	31	73 (62–86)	44 (12–76)	144 (93–194)	0·002	3·3 (2·2–4·4)	22·0 (13·2–30·8)
Zambia	2013	DHS	Ethnicity	47 987	32	70 (61–89)	27 (6–48)	220 (111–328)	<0·0001	8·2 (4·9–11·4)	31·4 (22·3–40·5)
Total	..	..	..	2 812 381	415	..	..	..	..	..	..

U5MR=Under-5 mortality rate. DHS=Demographic and Health Surveys. MICS=Multiple Indicator Cluster Survey.

* Calculated by comparing the the ethnic group with the highest mortality rate and lowest mortality rate in each country.

Mortality ratios and 95% CIs for the comparison between the ethnic groups with the highest and lowest mortality in each country are shown in the table. The median mortality ratio for all countries was 2·7 (IQR 1·8–4·6; range 1·4–10·0). In the 25 countries for which the likelihood ratio test p value was significant, the median mortality ratio was 3·3 (IQR 2·1–5·2; range 1·5–8·5) whereas among the remaining 11 countries with non-significant likelihood ratio test results, it was 1·9 (IQR 1·7–2·5; range 1·4–10·0). Only six of 36 countries (Benin, Guatemala, Guyana, India, Senegal, and South Africa) had 95% CIs for the U5MR ratio between the extreme groups that included unity, although the overall likelihood ratio test p value for Guatemala and India was significant. Large mortality ratios between extreme groups were observed in Ethiopia (mortality ratio 10·0, 95% CI 4·2–15·8) and Gabon (4·2, 95% CI 2·0–6·3), even in the absence of significant overall heterogeneity according to the likelihood ratio test.

Overall, across the 36 countries, the median Theil's index values were 28·9 for neonatal mortality, 40·1 for post-neonatal mortality, and 46·9 for child mortality (p=0·05 medians test; appendix p 20). The corresponding median values for between-group variance were 47·9 for neonatal mortality, 56·0 for post-neonatal mortality, and 104·8 for child mortality (p=0·03 medians test). These results suggest that ethnic inequalities increased with child age at death.

**Figure 1:**
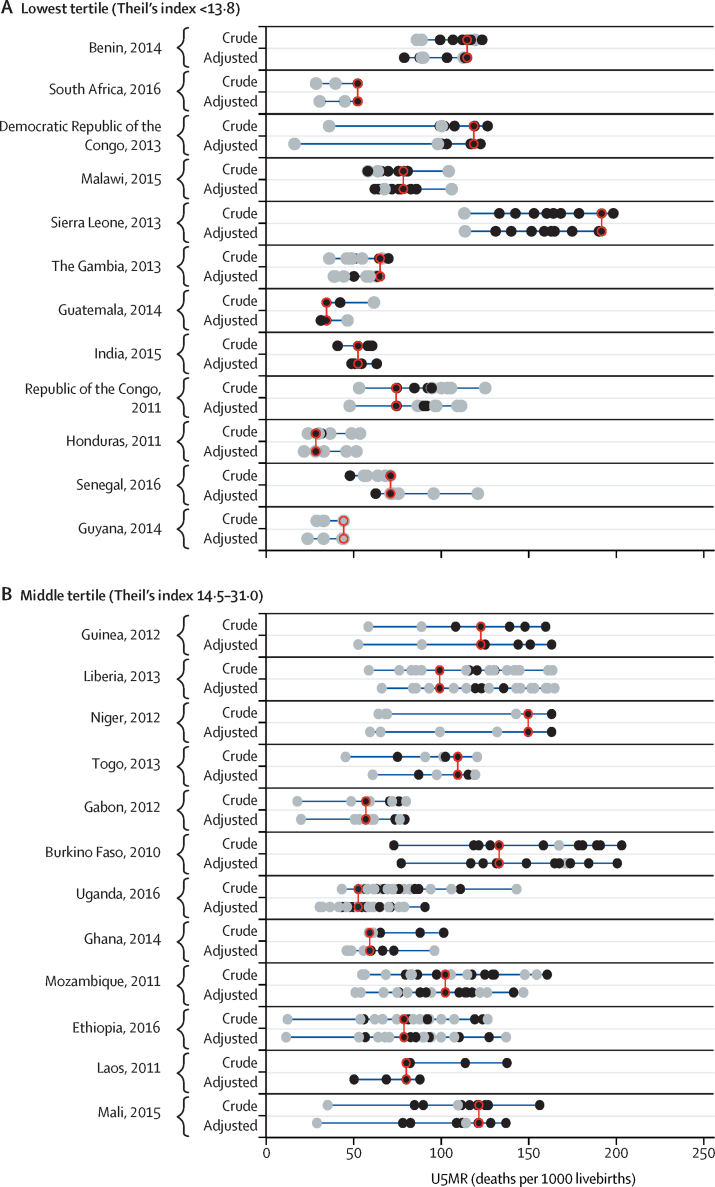

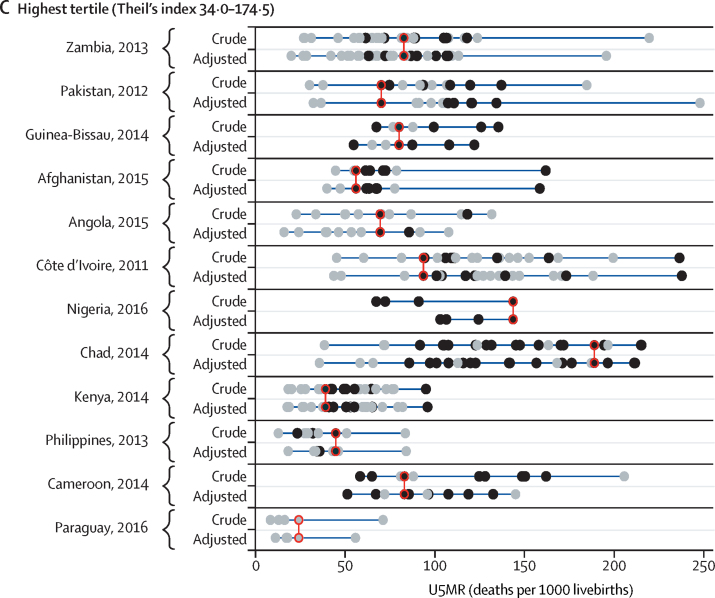
U5MR by ethnic group for countries in the lowest (A), middle (B), and highest (C) tertile of inequality according to Theil's index Black dots show estimates with a coefficient of variation <15% and grey dots show estimates with a coefficient of variation of ≥15%; red circles show the ethnic group with the largest number of births in each country. Estimates refer to the 10 years before the survey. Crude models included the child's age and ethnic group, and adjusted models also included household wealth, maternal education, and urban–rural residence. U5MR=under-5 mortality rate.

Figure 1 shows the U5MR for ethnic groups in the 36 countries according to Theil's index tertile. U5MRs for ethnic group by world regions are shown in the appendix (pp 21–23). In unadjusted analyses, Theil's index was less than 13·8 for countries in the lowest tertile (Benin, South Africa, Democratic Republic of the Congo, Malawi, Sierra Leone, The Gambia, Guatemala, India, Republic of the Congo, Honduras, Senegal, and Guyana; figure 1A), between 14·5 and 31·0 for countries in the middle tertile (Guinea, Liberia, Niger, Togo, Gabon, Burkina Faso, Uganda, Ghana, Mozambique, Ethiopia, Laos, and Mali; figure 1B), and between 34·0 and 174·5 for countries in the highest tertile (Zambia, Pakistan, Guinea-Bissau, Afghanistan, Angola, Cote d'Ivoire, Nigeria, Chad, Kenya, Philippines, Cameroon, and Paraguay; figure 1C). Overall U5MRs, neonatal, post-neonatal, and child mortality rates and 95% CIs, and crude and adjusted mortality ratios are shown in the appendix (pp 2–13).

For 15 ethnic groups, the lower bound of the 95% CI for U5MR was higher than 150 deaths per 1000 livebirths. These ethnic groups included the Nuristani in Afghanistan (U5MR estimate 162 deaths per 1000 livebirths); the Touareg-Bella (191 deaths per 1000 livebirths), Fulfuldé-Peul (181 deaths per 1000 livebirths), Sénoufo (179 deaths per 1000 livebirths), Gourmatché (189 deaths per 1000 livebirths), and Lobi (204 deaths per 1000 livebirths) in Burkina Faso; the Zarma in Niger (164 deaths per 1000 livebirths); the Temne (168 deaths per 1000 livebirths), Koranko (198 deaths per 1000 livebirths), and Mende (192 deaths per 1000 livebirths) in Sierra Leone; the Karo, Zimé, or Pévé (195 deaths per 1000 livebirths), Gabri, Kabalaye, Nangtchéré, or Soumray (215 deaths per 1000 livebirths), Sara (Ngambaye, Sara Madjin-Gaye, or Mbaye; 189 deaths per 1000 livebirths), and Baguirmi or Barma (215 deaths per 1000 livebirths) in Chad; and the Dioula in Côte d'Ivoire (237 deaths per 1000 livebirths). The group of ethnicities classified as other in Cameroon (206 deaths per 1000 livebirths) was also in the high mortality category.

Two groups had upper 95% CI bounds below the SDG target of 25 deaths per 1000 livebirths: the Spanish speakers in Paraguay (8 deaths per 1000 livebirths) and speakers of both Guarani and Spanish (16 deaths per 1000 livebirths).

In each of the remaining 27 countries, at least two ethnic groups had 95% CIs for U5MR that did not overlap with each other. For these countries, the mortality ratio between the extreme groups ranged from 1·8 (95% CI 1·0–2·5) in Guatemala (between the Maya and Ladino groups) to 10·0 (4·2–15·8) in Ethiopia (between the Gamo and Kembata groups).

In none of the countries studied did the largest ethnic group have the lowest U5MR (figure 1). In most countries, the mortality rate among the largest ethnic group was around the middle or in the upper half of the mortality distribution, and in two countries the largest group had the highest mortality (black individuals in South Africa and the Hausa in Nigeria).

**Figure 2:**
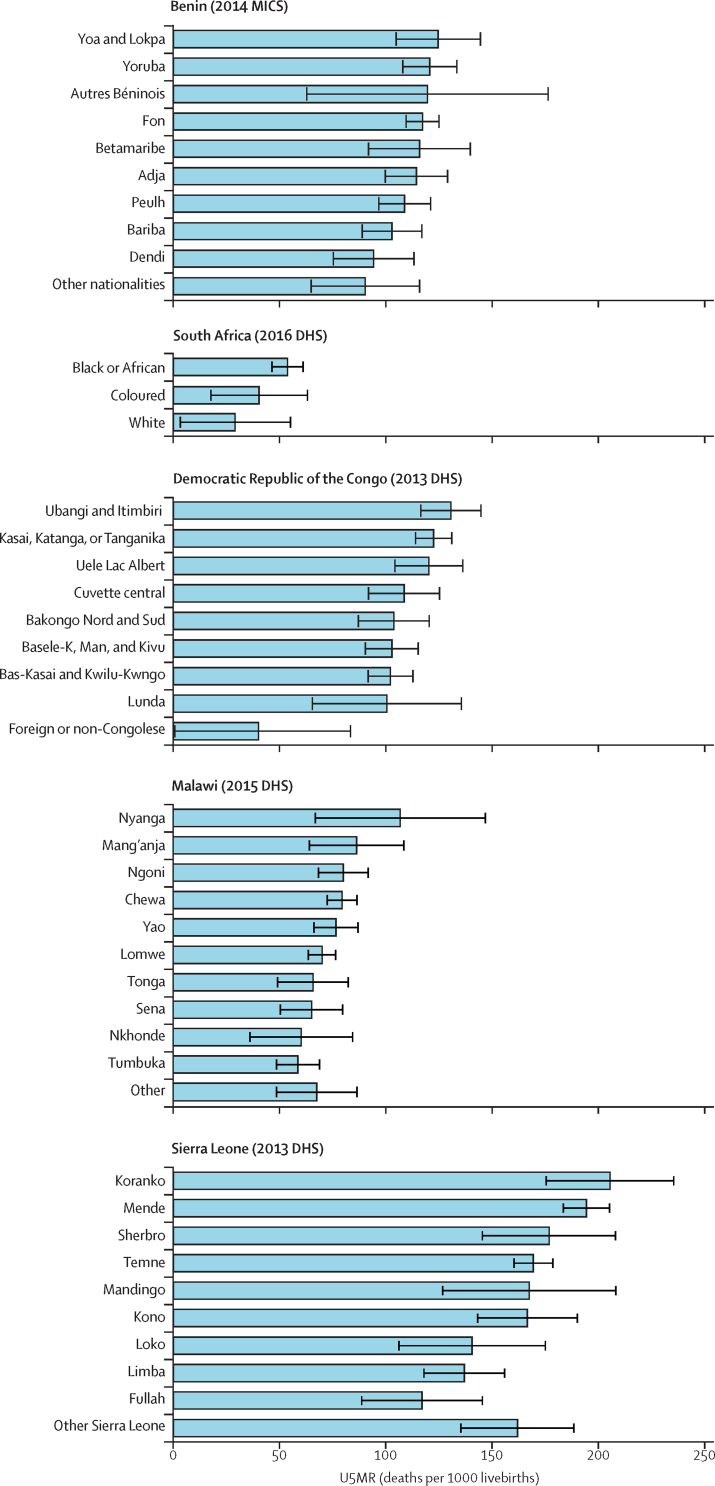
U5MR by ethnicity in the five countries with the narrowest relative inequalities (Benin, South Africa, Democratic Republic of the Congo, Malawi, and Sierra Leone), according to Theil's index Error bars show 95% CIs. U5MR=under-5 mortality rate. MICs= Multiple Indicator Cluster Surveys. DHS=Demographic and Health Surveys.

**Figure 3:**
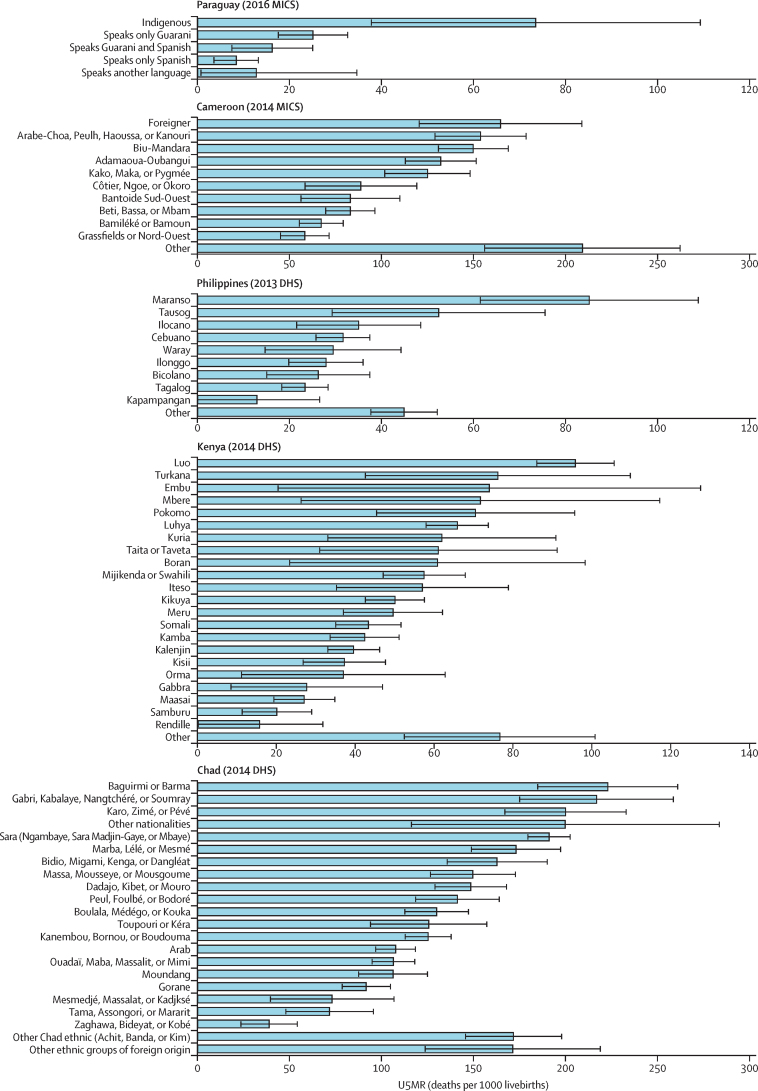
U5MR by ethnicity in the five countries with the widest relative inequalities (Paraguay, Cameroon, Philippines, Kenya, and Chad), according to Theil's index Error bars show 95% CIs. U5MR=under-5 mortality rate. MICs=Multiple Indicator Cluster Surveys. DHS=Demographic and Health Surveys.

Figure 2, Figure 3 show the unadjusted U5MR and 95% CIs for the five countries with the highest and lowest Theil's index values.

Results from the adjusted analyses are shown in figure 1, and full results are shown in the appendix (pp 2–13). In nearly all countries, adjustment for maternal education, household wealth, and urban–rural residence did not affect variability in mortality by ethnicity, with the exception of Nigeria, Laos, India, and Guatemala. In these four countries, the gaps in mortality between ethnic groups were substantially reduced after adjustment because substantial differences in wealth, education, and place of residence exist between ethnic groups in these countries, which could partly explain the ethnic gap in mortality. In Nigeria, the Hausa, who constituted the largest ethnic group, were markedly poorer than the rest of the sample and also had the highest mortality. In Laos, the largest ethnic group (the Lao people) were wealthier and had lower mortality than other ethnic groups. In Guatemala, the Maya and Xinca were poorer and had higher mortality than the largest ethnic group (the Ladinos or Mestizos). In India, scheduled tribes and castes had higher mortality and were poorer than the reference group (other backward classes).

## Discussion

To the best of our knowledge, this is the largest analysis of U5MR according to ethnicity to date, covering 415 ethnic groups in 36 countries. Our results show marked variability in U5MR by ethnic group; the median mortality ratio between the groups with the highest and lowest mortality rates in each country was equal to 2·7 in the 36 countries and 3·3 in the 25 countries with significant heterogeneity according to the likelihood ratio test. We also showed that the ethnic group with the largest number of births in each country was seldom the group with the lowest U5MR, suggesting that minorities—defined in terms of population size—were not necessarily being deprived of access to the services and resources required for survival of their children. We also showed that differences between ethnic groups tended to increase with child age at death, with greater disparities observed in deaths of children aged 1–4 years than for younger children, which has also been reported from high-income countries.^17^, ^18^, ^19^ Further research is needed to assess whether ethnic gaps in health intervention coverage also vary according to age of the children. Since neonatal and infant mortality rates are higher than those for children aged 1–4 years, the absolute gap between ethnic groups (expressed as differences between mortality rates) is greater for deaths in the first year than deaths after the first year of life.^20^

The wide ranging review of indigenous health by Anderson and colleagues^5^ documented higher infant mortality for indigenous children than non-indigenous children in 18 of 19 countries with data, but the authors did not attempt to discriminate between different indigenous groups. In 2000, Brockerhoff and Hewett^10^ analysed ethnic gaps in U5MRs in 11 countries in sub-Saharan Africa, by comparing one or two relatively privileged groups in each country with the rest of the national population. Consistent with our findings on ethnic disparities in most countries studied, in their analyses, statistically significant gaps were identified in all countries, except Mali. The authors did not attempt to compare multiple ethnicities within single countries.

Studies from the Philippines,^21^ China,^22^ and Pakistan^23^ have highlighted differences in coverage of child health interventions such as immunisation by ethnic group. However, few country-specific studies are available on mortality. Single country analyses of ethnic gaps in mortality are available for Brazil,^24^, ^25^ Cameroon,^26^ Nigeria,^27^ Mozambique,^28^ and Guinea-Bissau.^29^ These publications show substantial disparities according to ethnicity. In China,^22^ which was not included in the present analyses, minorities in the western regions had higher child mortality than did the dominant Han group. The association between caste and child mortality in India was reported in the 1990s, showing consistent disadvantages for children from scheduled castes and scheduled tribes,^30^, ^31^ which were consistent with our unadjusted results. In South Africa, we found a stepwise increase in U5MR from white individuals (U5MR 28 deaths per 1000 livebirths, 95% CI 3–54), to coloured individuals (39, 17–62) to black individuals (52, 45–60). The estimates for the first two categories had wide 95% CIs, and the summary indices for inequality were not statistically significant. However, other studies from this country reported wide and significant ethnic disparities.^32^, ^33^

The literature, which is mostly from high-income countries, suggests that adjustment for sociodemographic variables when comparing health outcomes between ethnic groups often shows that ethnic disparities are attenuated, but still persist.^34^ In the comparison of privileged ethnic groups and the rest of the population in 11 African countries,^10^ the authors concluded that adjustment for sociodemographic variables and household characteristics substantially reduced the survival advantage of privileged ethnic groups. In our analyses, ethnic gaps did not change substantially after adjustment for maternal education, household wealth, or urban–rural residence, with a few exceptions. This suggests that other mechanisms, including possibly discrimination affecting access to essential services and life-saving interventions, might account for the disparities observed. These differences could also partly reflect subnational variations in risk of mortality, since some ethnic groups are highly concentrated in specific areas. For example, in Kenya, more than 95% of the Kalenjin women interviewed in the 2014 DHS lived in the Rift Valley region.

Our analyses had limitations, which include the use of self-reported ethnicity or proxy variables; these issues also affect other studies of ethnic disparities in health.^5^ The method by which different ethnic groups were classified was dependent on the agencies that developed questionnaires for each country, which might not have used consistent approaches. This inconsistency is suggested by the wide variability in the number of ethnic groups among countries. Some groups were labelled as other, indicating national ethnic groups that were not listed separately, or as foreigners or none, indicating individuals who reported that they did not belong to a specific ethnic group, which was observed in Honduras, India, and Liberia. We also note that inconsistencies might exist between successive surveys in some countries—eg, the Nigeria 2013 DHS recognised 11 ethnic groups,^35^ whereas the 2016 MICS used in the present analyses pooled these 11 groups into four groups. Furthermore, some ethnic groups, such as nomads or individuals living in conflict-ridden areas, might be under-represented in the sample. Our results on the relative sizes of ethnic groups are based on number of births, rather than proportions of the population. For example, in Senegal, a higher number of births was recorded among the Poular than the Wolof; although the Wolof account for a larger proportion of the population than the Poular, they have lower fertility.^36^

Sample size considerations require the use of information on child deaths from the 10 years before the survey; this is a standard approach used in stratified analyses of mortality based on survey data, but as a consequence the results for a subgroup reflect mortality rates that are dated with an average reference date 5 years before the survey took place. Another limitation is the fact that the dates of the surveys ranged from 2010 (Burkina Faso) to 2016 (Ethiopia), and U5MR tends to decrease over time in most countries. It should also be noted that information collected on household wealth, maternal education, and income referred to the date of the interview, whereas information on child deaths was retrospective. One additional limitation is that the survival analysis methods used for U5MR implicitly assume that ethnic differentials by age are constant; however, our age-stratified analyses showed that gaps increased slightly with child age at death. However, the observed gaps were in the same direction for all age ranges, and the interpretation of results should not be substantially affected.

The number of ethnic categories recorded in the national surveys might affect statistical comparisons between extreme groups and values of the summary indices for inequality. Countries with many ethnic groups will tend to have higher values for these measures than those with few groups, and this finding must be taken into account when interpreting the results. This problem affects all summary measures of inequality for unordered categories, not only the two measures used in the present analyses.^14^, ^37^ In the presentation of results, we focused on Theil's index, a measure of relative inequality that is consistent with the rate ratio approach used in the multivariable analyses.

Our analyses are limited to countries with surveys since 2010 providing data both on ethnicity and birth histories. We examined surveys from more than 100 countries, of which 36 countries had data that could be included in the present analyses. Whether our results can be generalised to other LMICs is debatable, but the fact that most countries had significant ethnic gaps in U5MR suggests that such inequalities might also affect other countries.

The purpose of our analyses was to present a broad picture of ethnic inequalities in child survival on the basis of recent national surveys. We identified wide gaps in most countries studied. A detailed examination of the national contexts in which these inequalities exist is beyond the scope of the present analyses, but we hope that our results will motivate national researchers to explore these disparities and their determinants in more detail. Further research might include an examination of the drivers of inequalities in different countries, comparisons between countries with contrasting patterns of ethnic group inequalities, and stratified analyses of national samples, for example restricting the analyses to rural populations or poor people living in urban areas.

Studies of health inequalities benefit from the use of multiple stratification variables to characterise households and individuals. Wealth quintiles can be used to represent similar proportions of the population over time, and thus are useful to assess whether gaps are being reduced; however, these quintiles are not very useful for targeting interventions at specific groups. Data on geographical inequalities are useful for targeting interventions, but within the boundaries of a specific province or district important disparities might exist, which is observed for poor individuals living in urban areas within large cities. Our analyses show that ethnicity is an important contributor to inequality in many LMICs. Ethnicity is relatively easy to assess in surveys and in registration systems and might make an important contribution to targeting interventions.

20 years after the publication of the seminal article by Brockerhoff and Hewett^10^ on ethnic inequalities in 11 African countries, it is regrettable that little action has resulted from their conclusion that “strong and consistent results in this study support placing the notion of ethnicity at the forefront of theories and analyses of child mortality”. With the current SDG emphasis on “leaving no one behind”, we strongly advocate the need for greater attention to be given to recording ethnicity in surveys and also in routine administrative systems and health information systems to enable monitoring, guide targeting of interventions, and evaluate the equity impact of ongoing and future health interventions. The magnitude of ethnic inequities documented in our analyses highlights a violation of human rights that goes beyond child mortality. National governments should be made accountable for failing to ensure the basic right of survival for all of their children, regardless of ethnic affiliation.
