# Supplementary material for: Association between ethnicity and under-5 mortality: analysis of data from demographic surveys from 36 low-income and middle-income countries
Source: Lancet Glob Health. 2020 Feb 19;8(3):e352–61. doi: 10.1016/S2214-109X(20)30025-5 (PMC7034191; doi:10.1016/S2214-109X(20)30025-5)
Supplement: Supplementary appendix [file mmc1.pdf]

THE LANCET
Global Health

Supplementary appendix

This appendix formed part of the original submission and has been peer reviewed. We post it as supplied by the authors.

Supplement to: Victora CG, Barros AJD, Blumenberg C, et al. Association between ethnicity and under-5 mortality: analysis of data from demographic surveys from 36 low-income and middle-income countries. *Lancet Glob Health* 2020; **8:** e352–61.

Annex A. Mortality rates by ethnicity according to two alternative methods. Rates refer to the 10-year period preceding each survey.

Crude and adjusted under-five mortality rates and respective rate ratios by ethnic groups, estimated using the R procedure.

Mortality rates and standard errors by age and ethnic groups, estimated by DHS-*syncmrates* procedure.

Ethnicity	N	Share (%)	Crude U5MR	Adjusted U5MR	Crude analyses		Adjusted analyses [c]		Crude ratio between highest and lowest U5MR groups (95% CI)	Under-five					Neonatal		Post-neonatal		Infant		Child	
					U5MR ratio	95% CI	U5MR ratio	95% CI		Rate	s.e.	LL	UL	CV	Rate	s.e.	Rate	s.e.	Rate	s.e.	Rate	s.e.
Afghanistan (2015 - DHS) [a]									3.6													
Baloch	1,520	1.2%	55	47	0.89	0.40; 1.97	0.84	0.38; 1.88	(2.4; 4.8)	56	10	35	76	0.19	20	7	21	7	42	9	15	5
Hazara	11,278	9.0%	64	63	1.12	0.85; 1.48	1.13	0.86; 1.48		64	6	53	76	0.09	21	2	31	5	52	5	13	2
Nuristani	5,237	4.2%	162	159	3.00	2.32; 3.88	2.83	2.18; 3.67		164	7	150	178	0.04	41	5	79	4	120	6	50	4
Other	1,905	1.5%	45	40	0.75	0.45; 1.26	0.71	0.43; 1.16		45	9	28	62	0.20	24	6	14	5	39	8	7	3
Pashai	2,344	1.9%	79	78	1.47	1.01; 2.12	1.38	0.95; 2.01		79	12	55	103	0.15	34	8	32	7	66	11	14	4
**Pashtun**	52,382	41.7%	56	56	1.00		1.00			56	2	52	61	0.04	26	2	21	2	47	2	10	1
Tajik	39,344	31.3%	62	62	1.11	0.87; 1.40	1.10	0.90; 1.35		62	3	57	68	0.05	24	1	25	2	49	3	14	1
Turkmen	2,687	2.1%	73	67	1.38	1.01; 1.87	1.20	0.89; 1.61		73	7	58	87	0.10	24	6	40	6	63	8	10	3
Uzbek	8,791	7.0%	71	68	1.25	0.98; 1.60	1.21	0.96; 1.52		73	6	61	85	0.08	26	4	28	5	53	6	21	5
Angola (2015 - DHS) [b]									5.8													
Chokwe/kioko	3,944	9.4%	50	40	0.72	0.51; 1.01	0.57	0.40; 0.82	(3.2; 8.3)	51	6	40	62	0.11	11	2	21	4	32	5	20	3
Fiote	371	0.9%	50	39	0.67	0.35; 1.27	0.56	0.32; 1.00		53	17	20	86	0.32	14	7	16	6	30	9	24	16
Kikongo/ukongo	3,001	7.1%	57	46	0.84	0.58; 1.22	0.67	0.46; 0.98		61	7	47	74	0.11	23	4	13	3	36	5	25	5
Kimbundu	1,024	2.4%	132	108	1.95	1.34; 2.83	1.55	1.05; 2.28		128	18	94	163	0.14	40	11	44	10	84	16	49	11
Kwanhama	1,372	3.3%	75	59	1.14	0.62; 2.08	0.85	0.45; 1.59		77	10	57	97	0.13	28	6	20	6	48	8	30	8
Luvale	222	0.5%	23	16	0.30	0.10; 0.91	0.23	0.08; 0.70		23	13	0	48	0.56	4	4	0	0	4	4	19	12
Muhumbi	817	1.9%	115	92	1.82	0.99; 3.34	1.32	0.73; 2.39		121	22	78	163	0.18	32	12	42	12	74	19	50	16
Nganguela	1,153	2.7%	87	70	1.27	0.90; 1.78	1.00	0.70; 1.43		88	14	61	114	0.15	34	9	25	6	59	11	30	8
Nhaneca	1,077	2.6%	70	53	1.02	0.74; 1.41	0.77	0.54; 1.10		73	12	50	96	0.16	25	6	26	8	51	10	23	6
Other	484	1.2%	34	24	0.43	0.20; 0.91	0.35	0.16; 0.76		36	11	15	58	0.30	11	5	13	5	24	7	13	8
**Portuguese**	22,248	53.0%	70	70	1.00		1.00			71	4	64	78	0.05	21	2	23	2	44	3	28	2
Umbundu	6,286	15.0%	118	86	1.76	1.44; 2.15	1.23	0.95; 1.59		119	6	108	130	0.05	40	3	38	3	78	4	44	4
Benin (2014 - MICS) [a]									1.4													
Adja	8,342	18.5%	112	103	0.95	0.80; 1.12	0.90	0.76; 1.07	(1.0; 1.8)	114	7	99	128	0.07	33	3	27	3	60	4	57	5
Bariba	3,550	7.9%	99	88	0.87	0.66; 1.14	0.77	0.59; 0.99		102	7	88	116	0.07	25	4	27	4	52	6	53	6
Betamaribe	2,020	4.5%	115	88	0.96	0.74; 1.25	0.77	0.59; 1.00		115	12	91	139	0.11	20	5	30	5	50	7	68	9
Dendi	1,630	3.6%	89	89	0.78	0.53; 1.13	0.77	0.56; 1.08		94	10	75	112	0.10	22	5	32	8	54	9	42	7
**Fon**	15,641	34.6%	115	115	1.00		1.00			116	4	109	124	0.03	39	2	32	2	71	3	49	3
Foreigner	1,312	2.9%	86	90	0.75	0.50; 1.12	0.78	0.54; 1.14		90	13	64	115	0.14	34	7	20	6	54	11	38	10
Other	221	0.5%	119	113	1.04	0.63; 1.72	0.98	0.61; 1.57		119	29	62	175	0.24	41	15	44	22	85	24	37	22
Peulh	3,609	8.0%	107	79	0.92	0.69; 1.23	0.69	0.52; 0.91		108	6	96	120	0.06	27	4	44	4	71	5	39	5
Yoa & lokpa	3,197	7.1%	123	115	1.06	0.88; 1.29	1.00	0.83; 1.21		124	10	104	143	0.08	44	6	36	6	80	8	47	7
Yoruba	5,661	12.5%	117	114	1.00	0.83; 1.21	0.99	0.83; 1.19		120	6	107	132	0.05	44	4	30	4	73	6	50	4

Crude and adjusted under-five mortality rates and respective rate ratios by ethnic groups, estimated using the R procedure.

Mortality rates and standard errors by age and ethnic groups, estimated by DHS-*syncmrates* procedure.

Ethnicity	N	Share (%)	Crude U5MR	Adjusted U5MR	Crude analyses		Adjusted analyses [c]		Crude ratio between highest and lowest U5MR groups (95% CI)	Under-five					Neonatal		Post-neonatal		Infant		Child	
					U5MR ratio	95% CI	U5MR ratio	95% CI		Rate	s.e.	LL	UL	CV	Rate	s.e.	Rate	s.e.	Rate	s.e.	Rate	s.e.
Burkina Faso (2010 - DHS) [a]									2.8													
Bissa	1,926	3.4%	73	78	0.56	0.43; 0.73	0.58	0.45; 0.75	(2.0; 3.5)	74	9	57	92	0.12	32	5	23	6	55	7	21	6
Bobo	1,981	3.5%	122	124	0.91	0.72; 1.15	0.93	0.74; 1.18		123	9	106	141	0.07	45	7	31	6	76	8	51	8
Dagara	1,740	3.1%	159	149	1.17	0.95; 1.43	1.12	0.91; 1.38		162	14	135	190	0.09	37	7	70	11	107	12	63	10
Dioula	489	0.9%	168	172	1.21	0.87; 1.70	1.29	0.95; 1.74		166	23	121	211	0.14	28	14	47	14	75	17	98	18
Fulfuldé / Peul	5,052	9.0%	181	165	1.36	1.12; 1.64	1.24	1.03; 1.49		183	9	167	200	0.05	37	3	58	5	96	6	97	8
Gourmatché	4,004	7.1%	189	174	1.44	1.23; 1.68	1.30	1.11; 1.53		192	9	174	210	0.05	51	5	51	6	102	7	100	7
Gourounsi	2,302	4.1%	119	117	0.88	0.69; 1.13	0.88	0.69; 1.12		119	11	97	140	0.09	32	5	41	7	73	8	50	7
Lobi	2,561	4.6%	204	201	1.64	1.36; 1.99	1.50	1.24; 1.83		211	13	186	236	0.06	55	8	59	7	114	10	109	10
Mossi	28,600	50.9%	134	134	1.00		1.00			136	3	130	142	0.02	29	2	38	2	67	2	74	2
Other	3,274	5.8%	128	132	0.95	0.77; 1.16	0.99	0.81; 1.21		130	9	113	147	0.07	32	4	41	5	73	6	61	8
Sénoufo	3,070	5.5%	179	185	1.36	1.17; 1.59	1.38	1.19; 1.61		178	10	159	197	0.05	38	5	53	6	90	8	96	7
Touareg / bella	854	1.5%	191	168	1.42	1.19; 1.70	1.26	1.03; 1.53		192	20	153	231	0.10	37	10	73	11	110	15	92	16
Cameroon (2014 - MICS) [a]									3.5													
Adamaoua-Oubangui	2,539	9.7%	128	86	1.58	1.18; 2.11	1.03	0.75; 1.41	(2.5; 4.6)	132	10	113	151	0.07	31	5	43	6	74	7	63	8
Arabe_Choa/Peulh/Haoussa/Kanouri	3,013	11.5%	150	119	1.91	1.50; 2.42	1.43	1.08; 1.89		154	13	129	178	0.08	55	7	40	5	95	9	65	8
Bamiléké/Bamoun	4,473	17.1%	65	67	0.76	0.55; 1.06	0.81	0.59; 1.10		67	6	55	79	0.09	16	2	22	3	39	4	30	5
Bantoide Sud-Ouest	1,404	5.4%	81	72	0.96	0.65; 1.42	0.87	0.57; 1.31		83	14	56	110	0.16	30	9	28	9	58	13	27	7
Beti/Bassa/Mbam	4,720	18.0%	83	83	1.00		1.00			83	7	70	96	0.08	26	3	23	3	50	5	35	5
Biu-Mandara	3,450	13.2%	149	97	1.83	1.47; 2.29	1.16	0.89; 1.52		150	10	131	169	0.06	27	4	48	5	76	6	80	7
Côtier/Ngoe/Okoro	961	3.7%	88	96	1.09	0.66; 1.81	1.15	0.70; 1.90		89	15	58	119	0.17	40	13	27	7	67	14	23	7
Foreigner	701	2.7%	162	133	2.09	1.50; 2.93	1.59	1.13; 2.24		165	23	120	209	0.14	53	15	45	9	98	17	73	19
Grassfields/Nord-Ouest	2,802	10.7%	58	51	0.71	0.51; 0.98	0.62	0.45; 0.86		58	7	45	71	0.12	21	5	20	4	40	7	19	4
Kako/Maka/Pygmée	1,375	5.2%	125	108	1.56	1.15; 2.13	1.29	0.97; 1.73		125	12	102	148	0.09	39	6	44	6	83	8	46	8
Other	602	2.3%	206	145	2.72	1.66; 4.46	1.74	1.05; 2.89		209	27	156	262	0.13	80	19	54	11	135	19	86	23
Chad (2014 - DHS) [a]									5.6													
Arab	7,148	10.4%	105	101	0.53	0.43; 0.67	0.53	0.44; 0.65	(3.7; 7.5)	108	5	97	118	0.05	32	3	31	3	63	4	48	4
Baguirmi / barma	870	1.3%	215	211	1.11	0.79; 1.56	1.12	0.81; 1.54		223	19	185	261	0.09	67	15	56	12	123	18	114	14
Bidio / migami / kenga / dangléat	1,940	2.8%	158	157	0.85	0.64; 1.11	0.83	0.63; 1.09		163	14	135	190	0.09	36	7	61	7	96	9	73	11
Boulala / médégo / kouka	2,201	3.2%	129	123	0.65	0.48; 0.88	0.65	0.49; 0.86		130	9	113	147	0.07	29	5	56	7	85	8	49	6
Dadajo / kibet / mouro	2,969	4.3%	145	142	0.77	0.59; 0.99	0.75	0.59; 0.96		148	10	129	168	0.07	29	6	42	7	71	8	83	9
Foreigners	214	0.3%	197	187	1.00	0.56; 1.77	0.99	0.56; 1.74		200	43	116	283	0.21	23	12	36	18	59	22	149	40
Gabri / Kabalaye / Nangtchéré / Soumray	993	1.4%	215	212	1.13	0.92; 1.38	1.12	0.92; 1.36		217	21	175	259	0.10	42	8	55	11	97	13	133	19
Gorane	8,025	11.6%	92	86	0.45	0.36; 0.57	0.45	0.37; 0.56		92	7	79	105	0.07	24	3	28	4	52	5	42	5
Kanembou / bornou / boudouma	7,580	11.0%	123	116	0.61	0.48; 0.76	0.61	0.5; 0.75		125	6	113	138	0.05	29	3	35	3	64	4	65	5
Karo / zimé / pévé	1,009	1.5%	194	196	1.03	0.77; 1.38	1.04	0.78; 1.39		200	17	167	233	0.08	56	9	54	10	110	14	101	15
Marba / lélé / mesmé	1,944	2.8%	172	176	0.92	0.71; 1.20	0.93	0.72; 1.20		173	12	149	197	0.07	36	5	58	8	93	8	88	11
Massa / mousseye / mousgoume	2,466	3.6%	147	141	0.74	0.56; 0.99	0.75	0.57; 0.98		150	12	127	173	0.08	45	6	41	7	86	9	69	9
Mesmedjé / Massalat / Kadjksé	484	0.7%	72	58	0.32	0.16; 0.64	0.31	0.16; 0.61		73	17	39	107	0.23	25	9	18	7	42	11	32	11

Crude and adjusted under-five mortality rates and respective rate ratios by ethnic groups, estimated using the R procedure.

Ethnicity	N	Share (%)	Crude U5MR	Adjusted U5MR	Crude analyses: U5MR ratio	Crude analyses: 95% CI	Adjusted analyses [c]: U5MR ratio	Adjusted analyses [c]: 95% CI	Crude ratio between highest and lowest U5MR groups (95% CI)
Moundang	1,727	2.5%	108	108	0.53	0.38; 0.74	0.57	0.42; 0.78	
Other	515	0.7%	163	168	0.89	0.56; 1.42	0.89	0.56; 1.42	
Other chad ethnic (achit/banda/kim)	2,616	3.8%	170	171	0.90	0.70; 1.15	0.91	0.72; 1.14	
Ouadaï / maba / massalit / mimi	5,197	7.5%	105	97	0.53	0.42; 0.65	0.51	0.42; 0.63	
Peul / foulbé / bodoré	1,295	1.9%	132	120	0.65	0.49; 0.86	0.63	0.48; 0.83	
Sara (Ngambaye/Sara Madjin-Gaye/Mbaye)	13,898	20.1%	189	189	1.00		1.00		
Tama / assongori / mararit	1,273	1.8%	72	66	0.35	0.25; 0.50	0.35	0.25; 0.49	
Toupouri / kéra	1,001	1.5%	124	113	0.60	0.43; 0.84	0.60	0.43; 0.84	
Zaghawa / bideyat / kobé	1,991	2.9%	38	36	0.19	0.11; 0.30	0.19	0.12; 0.30	
Congo, Dem. Rep. (2013 - DHS) [a]									3.5
Bakongo nord & sud	4,250	7.2%	102	117	0.89	0.67; 1.17	0.99	0.76; 1.28	(2.2; 4.8)
Bas-kasai et kwilu-kwngo	8,892	15.0%	99	103	0.86	0.71; 1.03	0.87	0.72; 1.05	
Basele-K, Man. et Kivu	10,052	17.0%	100	98	0.87	0.72; 1.04	0.82	0.68; 0.99	
Cuvette central	5,914	10.0%	108	100	0.86	0.69; 1.07	0.85	0.67; 1.06	
Foreigner	273	0.5%	36	16	0.13	0.05; 0.35	0.14	0.05; 0.37	
Kasai, Katanga, Tanganika	16,953	28.6%	119	119	1.00		1.00		
Lunda	609	1.0%	100	98	0.76	0.48; 1.20	0.83	0.53; 1.28	
Ugangi et Itimbiri	7,155	12.1%	126	122	1.08	0.87; 1.34	1.03	0.83; 1.29	
Uele lac albert	4,976	8.4%	119	117	1.03	0.83; 1.29	0.99	0.78; 1.24	
Congo, Republic (2011 - DHS) [a]									2.4
Duma	447	1.4%	53	48	0.65	0.44; 0.97	0.64	0.44; 0.93	(1.6; 3.1)
Foreigner	2,540	8.0%	92	89	1.23	0.93; 1.63	1.20	0.90; 1.59	
Kongo	12,545	39.3%	74	74	1.00		1.00		
Makaa	204	0.6%	104	111	1.45	0.78; 2.69	1.50	0.84; 2.68	
Mbéré/mbéti/kélé	1,606	5.0%	106	109	1.49	0.95; 2.34	1.47	0.92; 2.35	
Mbochi	4,343	13.6%	85	92	1.15	0.87; 1.52	1.23	0.94; 1.63	
Oubanguiens	526	1.7%	95	87	1.17	0.66; 2.05	1.17	0.66; 2.07	
Punu	1,427	4.5%	100	97	1.34	0.88; 2.03	1.31	0.85; 2.00	
Pygmée	512	1.6%	125	96	1.61	1.04; 2.47	1.30	0.83; 2.02	
Sangha	2,244	7.0%	95	90	1.25	0.90; 1.75	1.21	0.87; 1.69	
Téké	5,294	16.6%	75	75	0.97	0.72; 1.31	1.00	0.74; 1.36	
Côte d'Ivoire (2011 - DHS) [a]									5.2
Abron	539	1.9%	146	171	1.70	1.06; 2.73	1.82	1.13; 2.94	(3.6; 6.9)
Agni	1,548	5.5%	106	117	1.15	0.82; 1.63	1.25	0.89; 1.77	
Akye ou attie	428	1.5%	153	171	1.56	0.96; 2.53	1.83	1.07; 3.10	
Baoule	5,380	19.1%	94	94	1.00		1.00		
Bete	652	2.3%	112	140	1.29	0.80; 2.07	1.49	0.93; 2.37	
Burkina-faso	2,957	10.5%	111	104	1.15	0.86; 1.52	1.11	0.83; 1.48	
Dioula	1,144	4.1%	236	238	2.57	1.83; 3.60	2.54	1.83; 3.53	

Mortality rates and standard errors by age and ethnic groups, estimated by DHS-*syncmrates* procedure.

Ethnicity	Under-five: Rate	Under-five: s.e.	Under-five: LL	Under-five: UL	Under-five: CV	Neonatal: Rate	Neonatal: s.e.	Post-neonatal: Rate	Post-neonatal: s.e.	Infant: Rate	Infant: s.e.	Child: Rate	Child: s.e.
Moundang	106	10	87	125	0.09	23	6	33	6	55	8	54	7
Other	171	24	124	219	0.14	50	11	62	15	112	20	66	14
Other chad ethnic (achit/banda/kim)	172	13	145	198	0.08	38	6	61	8	100	10	80	10
Ouadaï / maba / massalit / mimi	106	6	95	118	0.06	37	4	29	3	65	5	44	5
Peul / foulbé / bodoré	141	12	118	164	0.08	29	6	49	8	77	11	69	10
Sara (Ngambaye/Sara Madjin-Gaye/Mbaye)	191	6	179	202	0.03	42	2	61	4	103	5	98	5
Tama / assongori / mararit	72	12	48	95	0.17	29	7	24	6	53	11	20	6
Toupouri / kéra	126	16	94	157	0.13	41	9	29	7	70	12	60	11
Zaghawa / bideyat / kobé	39	8	23	54	0.20	11	6	7	2	18	6	21	6
Congo, Dem. Rep. (2013 - DHS) [a]													
Bakongo nord & sud	103	8	86	119	0.08	33	4	31	4	64	6	42	5
Bas-kasai et kwilu-kwngo	101	5	91	112	0.05	30	3	37	4	66	4	38	4
Basele-K, Man. et Kivu	102	6	90	114	0.06	36	4	29	4	65	5	40	5
Cuvette central	108	8	91	124	0.08	21	3	29	4	50	6	61	7
Foreigner	40	22	0	83	0.55	0	0	28	20	28	20	12	9
Kasai, Katanga, Tanganika	122	4	113	130	0.04	29	2	38	2	68	3	58	3
Lunda	100	18	65	134	0.18	42	14	32	11	74	17	28	8
Ugangi et Itimbiri	130	7	116	144	0.06	30	3	37	4	66	5	68	6
Uele lac albert	119	8	103	135	0.07	32	5	43	5	75	7	48	5
Congo, Republic (2011 - DHS) [a]													
Duma	54	15	25	82	0.27	7	6	13	7	20	9	34	12
Foreigner	93	12	71	116	0.12	19	3	35	8	54	9	41	8
Kongo	76	6	65	87	0.07	25	4	19	3	44	4	34	4
Makaa	99	28	43	155	0.29	17	13	63	23	79	26	21	12
Mbéré/mbéti/kélé	107	18	72	143	0.17	45	15	39	12	84	17	26	6
Mbochi	91	9	72	110	0.10	29	6	26	5	55	8	38	7
Oubanguiens	95	21	54	135	0.22	30	12	17	8	47	15	50	13
Punu	100	22	57	142	0.22	33	12	20	6	54	13	48	18
Pygmée	125	25	76	173	0.20	37	11	34	11	71	17	58	18
Sangha	96	12	72	119	0.13	19	9	32	5	50	11	47	7
Téké	74	9	57	92	0.12	19	5	21	3	40	6	35	7
Côte d'Ivoire (2011 - DHS) [a]													
Abron	147	24	100	193	0.16	102	21	21	8	123	23	27	11
Agni	107	12	84	130	0.11	53	8	27	7	80	11	29	6
Akye ou attie	150	28	95	204	0.19	89	22	38	16	127	27	26	12
Baoule	95	7	81	108	0.07	31	4	28	4	60	6	37	4
Bete	108	24	62	154	0.22	42	15	33	13	75	19	36	12
Burkina-faso	114	11	93	135	0.09	32	6	42	7	74	9	43	5
Dioula	233	20	195	272	0.08	79	13	57	12	136	17	113	14

Crude and adjusted under-five mortality rates and respective rate ratios by ethnic groups, estimated using the R procedure.

Ethnicity	N	Share (%)	Crude U5MR	Adjusted U5MR	Crude analyses: U5MR ratio	Crude analyses: 95% CI	Adjusted analyses [c]: U5MR ratio	Adjusted analyses [c]: 95% CI	Crude ratio between highest and lowest U5MR groups (95% CI)
Djimini	401	1.4%	60	48	0.53	0.19; 1.45	0.51	0.18; 1.47	
Gouro	615	2.2%	81	83	0.87	0.36; 2.08	0.89	0.39; 2.02	
Guere	616	2.2%	121	127	1.30	0.78; 2.15	1.36	0.85; 2.17	
Guinee	387	1.4%	136	143	1.41	0.85; 2.34	1.53	0.93; 2.51	
Koro	275	1.0%	169	166	1.88	1.02; 3.44	1.78	0.95; 3.32	
Koulango	940	3.3%	101	103	1.12	0.69; 1.80	1.10	0.67; 1.8	
Koyaka ou koyara	1,107	3.9%	124	136	1.37	0.86; 2.19	1.45	0.92; 2.28	
Lobi	487	1.7%	199	188	2.21	1.50; 3.26	2.01	1.35; 3.00	
Mahou ou mahouka	416	1.5%	121	131	1.29	0.77; 2.16	1.40	0.82; 2.39	
Mali	1,160	4.1%	109	122	1.22	0.83; 1.80	1.31	0.87; 1.96	
Malinke ou Maninka	2,097	7.4%	94	101	0.99	0.70; 1.41	1.08	0.76; 1.54	
Other	207	0.7%	142	147	1.59	0.77; 3.28	1.57	0.86; 2.87	
Senoufo	3,139	11.1%	164	173	1.80	1.36; 2.37	1.85	1.41; 2.43	
Tagouana	252	0.9%	45	44	0.44	0.17; 1.13	0.47	0.17; 1.25	
Wobe	303	1.1%	112	124	1.21	0.58; 2.51	1.32	0.61; 2.85	
Yacouba ou dan	1,267	4.5%	135	139	1.49	1.05; 2.12	1.49	1.04; 2.13	
Ethiopia (2016 - DHS) [a]									10.0
Affar	3,232	7.8%	120	111	1.51	1.13; 2.02	1.40	0.99; 1.98	(4.2; 15.8)
Amhara	7,486	18.1%	82	83	1.04	0.82; 1.32	1.05	0.82; 1.34	
Anyiwak	698	1.7%	85	95	1.02	0.61; 1.71	1.20	0.69; 2.08	
Berta	1,136	2.7%	124	128	1.58	1.11; 2.25	1.61	1.14; 2.29	
Gamo	454	1.1%	127	138	1.74	0.64; 4.71	1.74	0.65; 4.61	
Gedeo	451	1.1%	93	94	1.20	0.87; 1.66	1.18	0.85; 1.64	
Gumuz	765	1.8%	88	86	1.10	0.58; 2.1	1.09	0.57; 2.09	
Guragie	1,022	2.5%	67	71	0.86	0.6; 1.24	0.89	0.62; 1.28	
Hadiya	558	1.3%	94	91	1.12	0.74; 1.68	1.15	0.76; 1.73	
Kefficho	544	1.3%	100	100	1.22	0.66; 2.26	1.27	0.69; 2.32	
Kembata	289	0.7%	13	12	0.14	0.04; 0.55	0.15	0.04; 0.6	
Nuwer	966	2.3%	92	90	1.09	0.77; 1.54	1.14	0.74; 1.74	
Oromo	9,744	23.5%	79	79	1.00		1.00		
Shinasha	201	0.5%	108	108	1.35	0.68; 2.69	1.36	0.69; 2.7	
Sidama	1,019	2.5%	75	68	0.85	0.53; 1.37	0.86	0.53; 1.39	
Silte	364	0.9%	63	64	0.77	0.39; 1.53	0.81	0.42; 1.56	
Somalie	5,215	12.6%	93	86	1.14	0.9; 1.43	1.08	0.81; 1.44	
Tigrie	4,652	11.2%	56	57	0.69	0.53; 0.9	0.72	0.55; 0.94	
Welaita	698	1.7%	54	53	0.67	0.41; 1.09	0.68	0.42; 1.08	
Gabon (2012 - DHS) [a]									4.2
Fang	3,863	18.7%	71	80	1.29	0.93; 1.80	1.39	0.98; 1.98	(2.0; 6.3)
Kota-kele	2,616	12.6%	76	74	1.36	0.91; 2.05	1.29	0.87; 1.92	

Mortality rates and standard errors by age and ethnic groups, estimated by DHS-*syncmrates* procedure.

Ethnicity	Under-five: Rate	Under-five: s.e.	Under-five: LL	Under-five: UL	Under-five: CV	Neonatal: Rate	Neonatal: s.e.	Post-neonatal: Rate	Post-neonatal: s.e.	Infant: Rate	Infant: s.e.	Child: Rate	Child: s.e.
Djimini	60	19	23	98	0.32	22	14	12	10	34	17	27	14
Gouro	81	16	51	112	0.19	31	11	27	9	58	14	25	12
Guere	124	20	84	164	0.16	27	9	58	15	84	18	44	16
Guinee	138	27	86	190	0.19	22	9	41	18	64	19	79	23
Koro	175	26	124	226	0.15	25	14	61	19	86	23	98	26
Koulango	103	22	60	145	0.21	46	18	17	5	63	18	42	17
Koyaka ou koyara	123	19	86	160	0.15	52	15	31	10	83	14	43	13
Lobi	199	37	127	271	0.18	73	28	44	15	117	34	92	17
Mahou ou mahouka	128	25	80	177	0.19	33	15	40	17	73	22	60	16
Mali	109	14	82	137	0.13	42	8	37	9	79	13	33	8
Malinke ou Maninka	98	9	81	116	0.09	32	5	26	5	58	7	43	7
Other	146	33	81	212	0.23	37	16	18	12	55	20	97	27
Senoufo	166	10	146	187	0.06	70	9	46	6	116	10	57	7
Tagouana	46	17	12	79	0.38	8	7	0	0	8	7	38	16
Wobe	111	28	55	166	0.26	57	19	16	6	73	19	41	17
Yacouba ou dan	136	15	107	165	0.11	39	10	51	11	90	14	51	9
Ethiopia (2016 - DHS) [a]													
Affar	123	9	105	141	0.08	39	5	41	6	80	7	46	6
Amhara	82	6	70	93	0.07	46	5	18	3	65	6	18	3
Anyiwak	84	16	52	117	0.19	31	10	30	10	61	15	25	7
Berta	126	12	103	149	0.09	27	6	46	8	73	10	57	9
Gamo	132	25	83	181	0.19	23	9	59	17	82	18	54	21
Gedeo	95	17	62	128	0.18	4	4	45	12	48	13	49	13
Gumuz	87	16	55	119	0.19	41	9	10	5	52	11	38	11
Guragie	68	13	42	95	0.20	24	9	25	9	48	11	21	8
Hadiya	93	17	59	127	0.19	38	12	29	10	67	14	28	10
Kefficho	101	25	52	150	0.25	36	16	24	13	60	18	43	16
Kembata	12	9	0	30	0.71	11	9	0	0	11	9	1	1
Nuwer	91	15	62	119	0.16	26	8	12	4	38	10	55	13
Oromo	80	4	71	89	0.06	38	3	24	3	62	4	19	3
Shinasha	106	27	52	160	0.26	84	26	14	10	98	28	9	9
Sidama	76	12	52	101	0.16	34	9	23	7	57	11	20	7
Silte	62	16	29	94	0.27	39	16	14	9	54	18	9	6
Somalie	93	6	81	105	0.06	40	3	26	3	67	5	28	4
Tigrie	57	6	46	68	0.10	33	4	8	2	41	4	17	3
Welaita	56	13	29	82	0.24	31	9	20	8	51	13	4	4
Gabon (2012 - DHS) [a]													
Fang	76	9	57	94	0.12	29	5	23	5	52	8	25	5
Kota-kele	78	11	55	100	0.15	28	7	25	5	53	10	26	6

Crude and adjusted under-five mortality rates and respective rate ratios by ethnic groups, estimated using the R procedure.

Ethnicity	N	Share (%)	Crude U5MR	Adjusted U5MR	Crude analyses		Adjusted analyses [c]		Crude ratio between highest and lowest U5MR groups (95% CI)
					U5MR ratio	95% CI	U5MR ratio	95% CI	
Mbede-teke	1,521	7.3%	49	51	0.92	0.56; 1.52	0.89	0.53; 1.48	
Myene	640	3.1%	18	20	0.32	0.08; 1.22	0.36	0.09; 1.38	
Nzabi-duma	2,834	13.7%	72	77	1.31	0.82; 2.11	1.34	0.84; 2.15	
Okande-tsogho	933	4.5%	60	54	1.04	0.63; 1.71	0.94	0.58; 1.50	
Other	2,832	13.7%	59	60	1.05	0.71; 1.57	1.05	0.70; 1.57	
Pygmee	273	1.3%	80	62	1.40	0.65; 3.00	1.08	0.54; 2.16	
**Shira-punu/vili**	5,107	24.7%	57	57	1.00		1.00		
Gambia, The (2013 - DHS) [a]									1.9
Bambara	274	1.0%	36	39	0.56	0.21; 1.51	0.60	0.22; 1.58	(1.2; 2.7)
Foreigner	1,278	4.8%	66	63	0.99	0.68; 1.45	0.97	0.65; 1.45	
Fula/tukulur/lorobo	7,007	26.3%	65	57	0.95	0.70; 1.27	0.88	0.65; 1.18	
Jola/karoninka	2,049	7.7%	51	50	0.73	0.52; 1.02	0.77	0.55; 1.07	
**Mandinka/jahanka**	8,899	33.5%	65	65	1.00		1.00		
Manjago	279	1.0%	49	57	0.74	0.25; 2.16	0.88	0.30; 2.60	
Other	285	1.1%	46	39	0.62	0.25; 1.56	0.60	0.23; 1.57	
Serahuleh	1,770	6.7%	70	64	1.02	0.74; 1.40	0.98	0.72; 1.33	
Serere	843	3.2%	55	59	0.78	0.44; 1.37	0.91	0.51; 1.61	
Wollof	3,495	13.1%	49	44	0.71	0.47; 1.08	0.68	0.46; 1.02	
Ghana (2014 - DHS) [a]									1.7
**Akan**	8,986	38.9%	60	60	1.00		1.00		(1.2; 2.3)
Ewe	2,488	10.8%	66	60	1.07	0.78; 1.47	1.01	0.74; 1.39	
Ga/dangme	1,143	4.9%	61	56	0.99	0.64; 1.51	0.94	0.62; 1.44	
Grusi	1,007	4.4%	62	49	1.01	0.64; 1.57	0.83	0.51; 1.33	
Guan	641	2.8%	102	97	1.72	1.06; 2.78	1.62	1.01; 2.58	
Gurma	2,077	9.0%	102	73	1.74	1.35; 2.25	1.23	0.89; 1.70	
Mande	278	1.2%	60	47	1.02	0.44; 2.41	0.79	0.34; 1.83	
Mole-dagbani	6,109	26.4%	88	67	1.47	1.16; 1.85	1.12	0.85; 1.49	
Other	388	1.7%	59	46	0.98	0.49; 1.95	0.78	0.39; 1.54	
Guatemala (2014 - DHS) [a]									1.8
**Ladina/mestiza**	31,678	57.2%	34	34	1.00		1.00		(1.0; 2.5)
Maya	23,012	41.5%	42	31	1.21	1.01; 1.46	0.91	0.76; 1.09	
Xinca	610	1.1%	62	47	1.79	0.95; 3.38	1.35	0.73; 2.49	
Guinea (2012 - DHS) [a]									2.7
Guerzé	961	3.5%	89	89	0.73	0.49; 1.08	0.73	0.49; 1.08	(1.9; 3.5)
Kissi	1,472	5.3%	148	151	1.30	1.01; 1.67	1.23	0.94; 1.61	
Malinké	9,292	33.6%	160	163	1.34	1.12; 1.60	1.33	1.12; 1.57	
Other	581	2.1%	140	144	1.13	0.85; 1.49	1.17	0.88; 1.57	
**Peulh**	11,163	40.3%	123	123	1.00		1.00		
Soussou	3,955	14.3%	109	126	0.88	0.66; 1.17	1.02	0.77; 1.36	

Mortality rates and standard errors by age and ethnic groups, estimated by DHS-*syncmrates* procedure.

Ethnicity	Under-five					Neonatal		Post-neonatal		Infant		Child	
	Rate	s.e.	LL	UL	CV	Rate	s.e.	Rate	s.e.	Rate	s.e.	Rate	s.e.
Mbede-teke	54	12	31	77	0.22	10	4	14	4	23	6	31	10
Myene	18	14	0	45	0.74	12	13	3	1	15	13	4	2
Nzabi-duma	73	13	47	99	0.18	37	11	26	6	63	13	10	3
Okande-tsogho	61	13	34	87	0.22	8	4	24	6	32	6	30	11
Other	60	10	41	80	0.17	19	5	16	4	35	7	26	8
Pygmee	77	22	33	120	0.29	29	14	16	11	45	17	34	16
**Shira-punu/vili**	59	9	41	78	0.16	29	7	12	3	41	8	19	4
Gambia, The (2013 - DHS) [a]													
Bambara	35	14	7	63	0.41	31	14	3	3	34	14	1	1
Foreigner	66	12	42	90	0.19	31	9	16	5	47	10	19	6
Fula/tukulur/lorobo	65	6	54	76	0.09	26	3	14	3	40	4	26	4
Jola/karoninka	55	7	42	68	0.12	25	5	6	3	32	5	24	6
**Mandinka/jahanka**	66	5	56	76	0.08	34	4	12	2	46	4	21	2
Manjago	49	20	10	87	0.41	22	10	20	13	42	18	7	8
Other	46	31	0	107	0.67	2	2	0	0	2	2	44	31
Serahuleh	71	9	53	89	0.13	26	5	13	4	39	6	33	6
Serere	58	11	35	80	0.20	17	7	21	9	38	11	20	7
Wollof	49	8	34	65	0.16	15	3	16	5	30	6	19	6
Ghana (2014 - DHS) [a]													
**Akan**	60	4	53	67	0.06	33	3	13	2	46	3	14	2
Ewe	66	9	47	84	0.14	27	4	14	4	41	5	26	8
Ga/dangme	62	14	34	90	0.23	32	10	12	5	43	11	19	8
Grusi	58	16	28	89	0.27	32	14	7	2	40	14	19	6
Guan	101	16	70	132	0.16	47	14	33	9	81	15	22	9
Gurma	104	9	87	120	0.08	27	7	30	5	57	8	49	6
Mande	61	16	29	92	0.26	25	9	20	11	45	15	16	13
Mole-dagbani	90	8	75	106	0.09	29	4	22	4	51	6	42	6
Other	59	22	15	103	0.38	24	15	24	14	48	21	12	7
Guatemala (2014 - DHS) [a]													
**Ladina/mestiza**	35	2	31	39	0.06	16	1	12	1	28	2	7	1
Maya	43	2	39	46	0.04	19	1	13	1	32	2	11	1
Xinca	61	18	27	96	0.28	26	11	32	13	59	17	3	3
Guinea (2012 - DHS) [a]													
Guerzé	90	14	63	117	0.15	28	8	21	7	49	10	43	10
Kissi	153	15	124	183	0.10	30	7	48	7	78	11	82	13
Malinké	162	7	148	176	0.04	45	4	48	3	93	5	76	5
Other	141	25	92	189	0.17	48	12	10	4	58	14	87	19
**Peulh**	125	4	116	133	0.03	45	3	30	2	75	4	54	3
Soussou	110	7	96	123	0.06	31	4	28	3	59	5	54	5

Crude and adjusted under-five mortality rates and respective rate ratios by ethnic groups, estimated using the R procedure.

Ethnicity	N	Share (%)	Crude U5MR	Adjusted U5MR	Crude analyses: U5MR ratio	Crude analyses: 95% CI	Adjusted analyses [c]: U5MR ratio	Adjusted analyses [c]: 95% CI	Crude ratio between highest and lowest U5MR groups (95% CI)
Toma	259	0.9%	59	53	0.47	0.19; 1.14	0.43	0.18; 1.04	
Guinea-Bissau (2014 - MICS) [b]									2.0
Balanta	4,868	17.6%	68	55	0.82	0.64; 1.05	0.68	0.51; 0.92	(1.4; 2.6)
Crioulo	7,008	25.4%	80	80	1.00		1.00		
Fula	6,622	24.0%	136	122	1.79	1.46; 2.21	1.52	1.18; 1.97	
Mandinga	3,262	11.8%	126	108	1.62	1.26; 2.07	1.35	1.02; 1.79	
Manjaco	833	3.0%	88	73	1.05	0.73; 1.50	0.91	0.62; 1.33	
Other language	3,179	11.5%	99	87	1.28	0.99; 1.66	1.09	0.82; 1.46	
Papel	1,705	6.2%	77	65	0.97	0.60; 1.56	0.81	0.50; 1.33	
Guyana (2014 - MICS) [a]									1.5
African	2,728	24.4%	33	44	0.73	0.42; 1.27	0.98	0.55; 1.76	(0.8; 2.2)
Amerindian	2,489	22.3%	33	24	0.72	0.45; 1.15	0.53	0.31; 0.93	
East Indian	3,574	32.0%	44	44	1.00		1.00		
Mixed Race	2,331	20.9%	29	33	0.64	0.30; 1.37	0.74	0.33; 1.68	
Honduras (2011 - DHS) [a]									2.2
Garífuna	815	1.7%	37	33	1.13	0.51; 2.53	1.16	0.52; 2.57	(1.2; 3.3)
Lenca	4,794	9.7%	32	29	1.12	0.83; 1.50	1.02	0.75; 1.38	
Maya chorti	688	1.4%	24	22	0.78	0.35; 1.73	0.76	0.34; 1.71	
Misquito	1,919	3.9%	54	46	1.80	1.30; 2.48	1.61	1.15; 2.27	
Negro inglés	460	0.9%	49	52	1.66	0.74; 3.72	1.81	0.78; 4.19	
None	38,787	78.7%	28	28	1.00		1.00		
Other	1,430	2.9%	30	28	0.95	0.55; 1.65	0.99	0.56; 1.75	
India (2015 - DHS) [a]									1.5
Don't know	7,067	0.6%	58	63	1.33	1.08; 1.63	1.20	0.98; 1.48	(0.9; 2.1)
None of them	241,608	19.1%	41	49	0.77	0.73; 0.81	0.93	0.88; 0.98	
Other backward class	521,171	41.2%	53	53	1.00		1.00		
Schedule caste	246,873	19.5%	59	54	1.13	1.08; 1.18	1.04	0.99; 1.08	
Schedule tribe	248,330	19.6%	61	51	1.17	1.11; 1.24	0.96	0.91; 1.01	
Kenya (2014 - DHS) [a]									5.2
Boran	1,335	1.6%	62	60	1.52	0.59; 3.92	1.53	0.57; 4.11	(2.6; 7.8)
Embu	734	0.9%	73	82	1.98	0.71; 5.50	2.10	0.75; 5.88	
Gabbra	495	0.6%	28	26	0.70	0.37; 1.29	0.67	0.35; 1.27	
Iteso	888	1.1%	57	56	1.44	0.85; 2.43	1.43	0.85; 2.39	
Kalenjin	12,186	14.6%	39	39	1.00		1.00		
Kamba	7,128	8.5%	42	41	1.03	0.78; 1.36	1.05	0.80; 1.38	
Kikuya	10,899	13.0%	49	53	1.24	0.97; 1.59	1.36	1.05; 1.75	
Kisii	4,617	5.5%	36	38	0.93	0.67; 1.29	0.96	0.69; 1.34	
Kuria	691	0.8%	58	54	1.43	0.97; 2.12	1.38	0.93; 2.03	
Luhya	9,767	11.7%	65	65	1.63	1.29; 2.07	1.66	1.31; 2.11	

Mortality rates and standard errors by age and ethnic groups, estimated by DHS-*syncmrates* procedure.

Ethnicity	Under-five: Rate	Under-five: s.e.	Under-five: LL	Under-five: UL	Under-five: CV	Neonatal: Rate	Neonatal: s.e.	Post-neonatal: Rate	Post-neonatal: s.e.	Infant: Rate	Infant: s.e.	Child: Rate	Child: s.e.
Toma	62	24	14	110	0.40	19	12	21	14	39	18	24	16
Guinea-Bissau (2014 - MICS) [b]													
Balanta	67	5	57	77	0.07	23	4	13	2	36	5	32	3
Crioulo	81	5	71	91	0.06	40	4	18	3	58	5	24	4
Fula	138	5	128	148	0.04	55	3	30	3	85	4	57	4
Mandinga	128	9	110	146	0.07	46	5	33	5	79	7	53	6
Manjaco	96	14	68	124	0.15	39	9	13	5	52	11	46	12
Other language	105	9	88	122	0.08	44	6	21	4	65	7	43	6
Papel	81	9	64	98	0.11	15	4	23	5	39	6	44	7
Guyana (2014 - MICS) [a]													
African	33	6	20	46	0.19	18	5	12	4	29	6	4	2
Amerindian	33	5	23	43	0.16	9	3	15	4	24	5	9	3
East Indian	45	8	29	60	0.18	33	7	8	2	41	7	4	3
Mixed Race	30	9	12	47	0.30	14	7	8	3	22	7	8	5
Honduras (2011 - DHS) [a]													
Garífuna	36	16	5	68	0.44	17	12	11	8	28	15	9	6
Lenca	32	3	25	38	0.11	16	3	8	2	23	3	8	3
Maya chorti	24	7	11	38	0.28	20	7	2	1	22	7	2	2
Misquito	53	8	38	68	0.14	29	6	13	3	42	7	12	4
Negro inglés	48	17	14	83	0.36	16	9	22	12	38	14	11	11
None	29	1	26	32	0.05	16	1	7	1	23	1	6	1
Other	30	8	15	45	0.25	16	5	8	4	25	7	5	3
India (2015 - DHS) [a]													
Don't know	59	5	50	68	0.08	32	3	14	3	46	4	14	3
None of them	41	1	39	43	0.02	25	1	9	0	34	1	7	1
Other backward class	53	1	52	54	0.06	31	0	12	0	43	1	10	0
Schedule caste	59	1	57	61	0.01	34	1	13	0	47	1	13	0
Schedule tribe	61	1	58	63	0.02	33	1	14	1	46	1	15	1
Kenya (2014 - DHS) [a]													
Boran	61	19	23	98	0.31	26	15	26	10	52	19	9	4
Embu	74	27	21	128	0.37	59	27	0	0	59	27	16	7
Gabbra	28	10	9	47	0.35	17	8	4	4	22	10	6	7
Iteso	57	11	35	79	0.19	16	5	25	8	41	9	17	5
Kalenjin	40	3	33	46	0.08	21	2	13	2	34	3	6	1
Kamba	42	4	34	51	0.11	26	3	10	2	36	4	7	2
Kikuya	50	4	43	58	0.08	25	3	17	2	41	3	9	2
Kisii	37	5	27	48	0.14	21	4	10	3	31	5	7	2
Kuria	62	15	33	91	0.24	30	11	16	8	46	13	16	7
Luhya	66	4	58	74	0.06	24	3	20	2	44	4	23	2

Crude and adjusted under-five mortality rates and respective rate ratios by ethnic groups, estimated using the R procedure.

Ethnicity	N	Share (%)	Crude U5MR	Adjusted U5MR	Crude analyses: U5MR ratio	Crude analyses: 95% CI	Adjusted analyses [c]: U5MR ratio	Adjusted analyses [c]: 95% CI	Crude ratio between highest and lowest U5MR groups (95% CI)
Luo	8,814	10.5%	95	96	2.44	2.00; 2.98	2.45	2.01; 3.00	
Maasai	2,206	2.6%	25	27	0.70	0.43; 1.15	0.69	0.41; 1.16	
Mbere	355	0.4%	58	61	1.63	0.88; 3.02	1.57	0.83; 2.96	
Meru	3,332	4.0%	50	51	1.25	0.90; 1.74	1.29	0.93; 1.79	
Mijikenda / Swahili	4,798	5.7%	55	55	1.45	1.13; 1.86	1.41	1.08; 1.84	
Orma	339	0.4%	35	31	0.82	0.33; 2.02	0.80	0.32; 1.97	
Other	1,824	2.2%	77	79	1.95	1.32; 2.88	2.03	1.35; 3.06	
Pokomo	996	1.2%	67	65	1.77	1.05; 2.98	1.66	0.98; 2.82	
Rendille	287	0.3%	18	18	0.47	0.11; 2.01	0.45	0.11; 1.94	
Samburu	2,204	2.6%	20	19	0.50	0.31; 0.79	0.48	0.30; 0.79	
Somali	6,224	7.4%	43	44	1.13	0.84; 1.52	1.11	0.77; 1.61	
Taita / Taveta	1,079	1.3%	61	62	1.47	0.83; 2.61	1.59	0.90; 2.83	
Turkana	2,373	2.8%	76	71	1.89	1.33; 2.70	1.81	1.18; 2.77	
Lao PDR (2011 - MICS) [a]									1.7
Hmong	5,686	10.0%	83	51	1.03	0.85; 1.24	0.63	0.52; 0.77	(1.2; 2.2)
Khmu	8,184	14.4%	114	69	1.47	1.24; 1.74	0.86	0.72; 1.03	
Lao	23,127	40.7%	80	80	1.00		1.00		
Other	19,713	34.7%	138	88	1.78	1.53; 2.07	1.10	0.94; 1.28	
Liberia (2013 - DHS) [b]									2.7
Bassa	4,175	13.6%	117	120	1.21	0.90; 1.61	1.20	0.91; 1.59	(1.9; 3.5)
Belle	274	0.9%	164	165	1.64	0.87; 3.11	1.66	0.87; 3.18	
Gbandi	643	2.1%	86	108	1.05	0.57; 1.91	1.08	0.59; 1.99	
Gio	1,502	4.9%	83	86	0.83	0.54; 1.28	0.87	0.56; 1.35	
Gola	1,764	5.7%	131	136	1.33	1.03; 1.72	1.37	1.06; 1.76	
Grebo	4,840	15.7%	121	124	1.18	0.96; 1.47	1.24	1.01; 1.53	
Kissi	956	3.1%	89	94	0.90	0.61; 1.34	0.94	0.63; 1.41	
Kpelle	6,415	20.8%	100	100	1.00		1.00		
Krahn	1,708	5.5%	115	115	1.11	0.80; 1.55	1.15	0.83; 1.60	
Kru	2,433	7.9%	143	154	1.48	0.91; 2.39	1.54	0.98; 2.44	
Lorma	977	3.2%	128	128	1.21	0.72; 2.03	1.28	0.77; 2.14	
Mandingo	503	1.6%	142	152	1.45	1.04; 2.02	1.53	1.09; 2.13	
Mano	1,516	4.9%	76	84	0.81	0.51; 1.27	0.85	0.55; 1.31	
Mende	610	2.0%	131	146	1.40	0.91; 2.18	1.46	0.96; 2.23	
None/only English	248	0.8%	161	161	1.28	0.71; 2.31	1.62	0.87; 3.01	
Other	256	0.8%	59	67	0.59	0.39; 0.91	0.67	0.41; 1.09	
Sarpo	477	1.5%	138	143	1.37	0.75; 2.53	1.44	0.77; 2.71	
Vai	1,416	4.6%	145	160	1.53	1.07; 2.19	1.61	1.14; 2.27	
Malawi (2015 - DHS) [a]									1.8
Chewa	20,426	30.0%	78	78	1.00		1.00		(1.2; 2.4)

Mortality rates and standard errors by age and ethnic groups, estimated by DHS-*syncmrates* procedure.

Ethnicity	Under-five: Rate	Under-five: s.e.	Under-five: LL	Under-five: UL	Under-five: CV	Neonatal: Rate	Neonatal: s.e.	Post-neonatal: Rate	Post-neonatal: s.e.	Infant: Rate	Infant: s.e.	Child: Rate	Child: s.e.
Luo	96	5	86	106	0.05	16	2	40	3	56	4	42	3
Maasai	27	4	19	35	0.15	15	3	7	3	22	4	5	2
Mbere	72	23	26	117	0.32	11	6	12	6	24	8	49	22
Meru	50	6	37	62	0.13	28	5	10	3	38	6	12	3
Mijikenda / Swahili	57	5	47	68	0.09	24	3	19	3	43	5	15	3
Orma	37	13	11	63	0.36	28	12	0	0	28	12	9	7
Other	77	12	52	101	0.16	38	10	12	5	51	11	28	9
Pokomo	71	13	45	96	0.18	23	7	18	6	41	10	30	9
Rendille	16	8	0	32	0.52	12	7	2	3	15	8	1	1
Samburu	20	4	11	29	0.22	8	3	7	2	15	4	5	2
Somali	43	4	35	52	0.10	25	3	11	2	36	4	8	1
Taita / Taveta	61	15	31	91	0.25	18	6	33	13	51	14	11	5
Turkana	76	17	43	110	0.22	27	8	20	9	47	13	30	12
Lao PDR (2011 - MICS) [a]													
Hmong	85	5	76	94	0.05	29	2	34	4	63	4	23	2
Khmu	117	5	107	127	0.04	53	4	35	3	89	5	31	3
Lao	82	3	75	88	0.04	32	2	40	2	72	3	10	1
Other	140	5	131	149	0.03	55	3	58	3	113	4	31	2
Liberia (2013 - DHS) [b]													
Bassa	116	10	98	135	0.08	37	5	41	7	78	8	42	5
Belle	161	37	88	234	0.23	27	14	60	29	86	28	82	30
Gbandi	91	24	44	139	0.27	50	18	32	13	82	25	10	5
Gio	85	14	59	112	0.16	22	9	31	8	52	12	35	7
Gola	131	14	104	158	0.11	39	7	51	11	90	13	45	8
Grebo	124	12	101	148	0.10	45	5	35	8	80	9	48	6
Kissi	92	17	58	126	0.19	29	10	34	13	63	16	31	9
Kpelle	102	6	91	114	0.06	25	2	31	3	56	4	49	4
Krahn	115	14	88	142	0.12	18	4	40	6	58	8	60	13
Kru	144	18	109	178	0.12	48	13	44	8	92	12	57	12
Lorma	130	23	85	174	0.18	55	15	30	11	85	18	48	19
Mandingo	138	27	86	191	0.19	68	19	26	11	94	23	49	17
Mano	80	13	54	106	0.16	26	10	21	4	47	12	35	8
Mende	133	23	89	177	0.17	28	11	61	13	89	15	48	16
None/only English	161	37	88	234	0.23	29	18	30	15	59	22	108	30
Other	59	32	0	121	0.54	18	13	36	30	54	31	5	3
Sarpo	137	38	63	211	0.28	65	28	26	10	91	28	50	27
Vai	149	16	118	180	0.11	36	7	56	9	91	11	63	14
Malawi (2015 - DHS) [a]													
Chewa	79	4	72	86	0.05	28	2	19	2	47	3	34	2

Crude and adjusted under-five mortality rates and respective rate ratios by ethnic groups, estimated using the R procedure.

Mortality rates and standard errors by age and ethnic groups, estimated by DHS-*syncmrates* procedure.

Ethnicity	N	Share (%)	Crude U5MR	Adjusted U5MR	Crude analyses		Adjusted analyses [c]		Crude ratio between highest and lowest U5MR groups (95% CI)	Under-five					Neonatal		Post-neonatal		Infant		Child	
					U5MR ratio	95% CI	U5MR ratio	95% CI		Rate	s.e.	LL	UL	CV	Rate	s.e.	Rate	s.e.	Rate	s.e.	Rate	s.e.
Lomwe	12,219	17.9%	70	72	0.90	0.78; 1.04	0.92	0.79; 1.06		70	3	64	76	0.05	29	2	18	2	46	3	25	2
Mang'anja	1,724	2.5%	81	86	1.07	0.79; 1.43	1.09	0.82; 1.47		86	11	64	108	0.13	17	4	40	9	57	9	31	7
Ngoni	8,367	12.3%	79	83	1.01	0.83; 1.24	1.05	0.87; 1.28		80	6	68	92	0.07	28	3	26	3	53	5	28	3
Nkhonde	890	1.3%	58	68	0.79	0.52; 1.20	0.86	0.57; 1.31		60	12	36	84	0.20	27	8	9	5	35	9	26	6
Nyanga	1,441	2.1%	104	106	1.34	0.88; 2.06	1.35	0.88; 2.07		107	20	67	147	0.19	55	14	16	6	70	16	39	12
Other	2,352	3.5%	66	77	0.92	0.69; 1.23	0.98	0.73; 1.32		68	10	49	86	0.14	25	6	25	6	50	7	19	6
Sena	3,393	5.0%	64	64	0.80	0.62; 1.04	0.82	0.63; 1.07		65	7	50	80	0.11	24	4	14	3	38	6	28	5
Tonga	2,365	3.5%	64	68	0.81	0.58; 1.14	0.86	0.61; 1.21		66	8	49	82	0.13	19	4	15	4	34	5	33	7
Tumbuka	7,030	10.3%	58	62	0.74	0.60; 0.92	0.79	0.63; 0.99		59	5	49	69	0.09	24	3	16	2	40	4	19	3
Yao	7,867	11.6%	76	76	0.97	0.80; 1.17	0.97	0.80; 1.16		77	5	66	87	0.07	23	3	21	3	44	4	34	4
Mali (2015 - MICS) [a]									4.4													
Arabe/Maure/Tamacheq	2,159	3.9%	85	79	0.71	0.53; 0.95	0.64	0.47; 0.88	(2.8; 6.0)	88	9	70	107	0.11	41	6	21	5	62	8	28	6
Bambara/Malinké	20,875	37.4%	122	122	1.00		1.00			124	3	117	130	0.03	33	2	29	2	62	3	66	3
Bobo	1,048	1.9%	121	113	1.01	0.76; 1.35	0.92	0.70; 1.22		123	15	93	152	0.12	31	10	19	5	49	10	77	12
Bozo	1,247	2.2%	157	137	1.29	0.95; 1.75	1.13	0.83; 1.53		164	14	136	191	0.09	56	9	27	7	84	11	88	11
Dafing/Minianka/Samogo/Sénoufo	4,720	8.5%	127	122	1.04	0.83; 1.31	1.00	0.81; 1.22		131	7	118	144	0.05	37	4	26	3	62	5	73	6
Dogon	4,174	7.5%	36	30	0.28	0.16; 0.51	0.24	0.14; 0.43		37	5	27	46	0.13	7	2	12	3	19	3	19	4
Haoussa/Other	1,522	2.7%	112	113	0.92	0.71; 1.18	0.92	0.72; 1.19		111	13	86	137	0.12	32	6	27	5	60	8	55	10
Kassonké	493	0.9%	110	115	0.90	0.46; 1.77	0.94	0.48; 1.85		110	18	75	145	0.16	33	13	36	11	69	16	44	12
Peulh/Foulfoulbé	8,218	14.7%	117	109	0.96	0.80; 1.15	0.90	0.76; 1.06		119	5	110	128	0.04	34	3	28	3	62	4	61	4
Sarakolé/Soninké/Marka	6,737	12.1%	126	129	1.04	0.84; 1.28	1.06	0.86; 1.30		126	7	113	139	0.05	41	4	27	3	68	5	62	6
Sonraï/Djerma	4,579	8.2%	90	83	0.72	0.54; 0.95	0.68	0.51; 0.91		93	9	74	111	0.10	37	6	19	3	56	7	39	6
Mozambique (2011 - DHS) [a]									2.9													
Bitonga	746	2.0%	55	51	0.52	0.30; 0.93	0.50	0.28; 0.88	(2.0; 3.8)	56	13	30	81	0.23	22	10	16	6	38	9	18	10
Chitewe	821	2.2%	131	122	1.32	0.96; 1.83	1.19	0.85; 1.67		129	19	92	165	0.15	21	8	65	14	85	16	47	12
Cibalke	389	1.0%	155	147	1.55	1.09; 2.20	1.43	0.98; 2.10		153	29	96	210	0.19	21	10	40	14	61	14	98	28
Cicewa	2,510	6.6%	125	114	1.25	0.96; 1.62	1.11	0.84; 1.47		127	10	107	147	0.08	43	5	36	5	79	7	52	8
Cichopi	909	2.4%	57	55	0.54	0.34; 0.87	0.53	0.33; 0.85		59	12	36	82	0.20	12	5	32	9	44	10	16	8
Cindau	2,195	5.8%	115	111	1.15	0.89; 1.50	1.08	0.81; 1.43		119	10	99	140	0.09	30	6	40	6	70	8	53	7
Cinyungwe	1,208	3.2%	118	115	1.23	0.90; 1.69	1.12	0.80; 1.56		117	13	91	143	0.11	49	11	47	7	95	13	24	6
Cisena	4,295	11.3%	98	88	0.98	0.78; 1.23	0.86	0.65; 1.13		101	9	83	120	0.09	40	5	32	6	73	8	31	5
Ciyao	1,375	3.6%	106	95	1.04	0.72; 1.51	0.92	0.61; 1.38		106	12	82	129	0.11	33	6	41	7	74	9	35	8
Echuwabo	1,581	4.2%	130	118	1.29	1.00; 1.67	1.15	0.87; 1.52		132	13	107	158	0.10	39	7	46	7	86	9	51	11
Elomwe	2,241	5.9%	161	142	1.64	1.30; 2.08	1.38	1.04; 1.82		162	14	135	189	0.09	37	5	68	7	105	9	64	11
Emakhuwa	6,321	16.6%	87	77	0.86	0.70; 1.05	0.74	0.59; 0.95		88	5	78	98	0.06	19	2	38	3	57	4	33	3
Other	384	1.0%	148	127	1.36	0.93; 1.98	1.23	0.80; 1.90		149	35	80	217	0.24	33	16	86	26	119	32	33	16
Portuguese	1,947	5.1%	80	92	0.79	0.58; 1.08	0.89	0.64; 1.24		81	9	63	99	0.11	35	6	23	5	58	7	25	6
Shimakonde	526	1.4%	84	75	0.80	0.42; 1.54	0.73	0.38; 1.41		83	14	56	110	0.17	41	11	14	5	55	11	30	11

Crude and adjusted under-five mortality rates and respective rate ratios by ethnic groups, estimated using the R procedure.

Mortality rates and standard errors by age and ethnic groups, estimated by DHS-*syncmrates* procedure.

Ethnicity	N	Share (%)	Crude U5MR	Adjusted U5MR	Crude analyses		Adjusted analyses [c]		Crude ratio between highest and lowest U5MR groups (95% CI)	Under-five					Neonatal		Post-neonatal		Infant		Child	
					U5MR ratio	95% CI	U5MR ratio	95% CI		Rate	s.e.	LL	UL	CV	Rate	s.e.	Rate	s.e.	Rate	s.e.	Rate	s.e.
Shona	744	2.0%	83	81	0.80	0.51; 1.26	0.79	0.50; 1.25		83	14	57	110	0.16	14	5	25	8	39	10	46	11
Xichangana	7,270	19.1%	103	103	1.00		1.00			103	5	94	113	0.05	37	3	30	3	67	4	39	3
Xirhonga	310	0.8%	115	123	1.17	0.60; 2.29	1.19	0.61; 2.33		116	29	58	173	0.25	44	19	39	16	83	24	35	19
Xitswa	2,105	5.5%	69	67	0.68	0.49; 0.94	0.66	0.47; 0.92		70	8	54	86	0.12	23	4	24	6	47	7	24	5
Niger (2012 - DHS) [b]									2.5													
French	339	0.8%	65	100	0.41	0.21; 0.78	0.66	0.34; 1.30	(1.8; 3.3)	65	22	21	108	0.34	16	10	15	9	31	13	35	19
Fulfuldé	497	1.1%	143	132	0.92	0.63; 1.34	0.88	0.60; 1.30		149	24	102	197	0.16	13	7	16	7	29	9	124	22
Houssa	29,178	66.0%	150	150	1.00		1.00			154	3	147	160	0.02	32	1	34	2	66	2	94	2
Kanouri/Toubou	2,186	4.9%	70	66	0.46	0.31; 0.70	0.44	0.29; 0.67		71	9	53	90	0.13	11	4	20	6	31	8	42	8
Tamasheq	985	2.2%	69	60	0.39	0.24; 0.64	0.40	0.24; 0.65		69	11	46	91	0.17	35	8	10	4	45	9	25	7
Zarma	10,646	24.1%	163	164	1.04	0.92; 1.17	1.09	0.97; 1.23		167	7	154	180	0.04	33	2	35	3	67	4	107	5
Nigeria (2016 - MICS) [a]									2.1													
Hausa	49,685	48.9%	144	144	1.00		1.00		(1.5; 2.8)	147	3	142	152	0.02	41	1	39	1	80	2	73	2
Igbo	10,548	10.4%	67	103	0.47	0.40; 0.55	0.72	0.60; 0.86		67	3	61	74	0.05	29	3	19	2	48	3	20	2
Other	31,459	30.9%	91	107	0.63	0.56; 0.71	0.74	0.66; 0.84		92	3	87	98	0.03	34	2	28	1	62	2	32	2
Yoruba	9,999	9.8%	72	124	0.50	0.43; 0.58	0.87	0.73; 1.03		74	5	64	83	0.07	37	3	19	2	57	5	18	2
Pakistan (2012 - DHS) [b]									6.1													
Balochi	2,266	4.5%	137	134	2.03	1.48; 2.79	1.92	1.41; 2.61	(3.8; 8.5)	134	22	90	178	0.17	83	18	16	6	98	19	40	14
Balti	1,585	3.2%	107	106	1.55	0.97; 2.49	1.51	0.92; 2.47		108	14	80	136	0.13	49	10	38	8	86	12	24	8
Barauhi	2,787	5.5%	137	134	2.05	1.68; 2.52	1.92	1.55; 2.37		140	11	119	161	0.08	70	8	47	7	116	9	26	6
Brushaski	436	0.9%	30	32	0.43	0.21; 0.89	0.46	0.21; 1.02		30	16	0	61	0.52	14	11	7	7	21	13	9	10
Chitrali/khwar	437	0.9%	38	37	0.53	0.28; 1.02	0.52	0.26; 1.04		38	20	0	77	0.52	8	4	27	19	34	19	4	3
Hindko	1,814	3.6%	82	98	1.22	0.70; 2.11	1.40	0.80; 2.44		81	12	58	103	0.14	63	11	11	4	75	12	6	4
Marwari	317	0.6%	98	90	1.34	0.75; 2.39	1.28	0.68; 2.40		97	23	52	142	0.24	55	21	10	7	65	22	34	14
Other	1,445	2.9%	94	104	1.37	0.91; 2.08	1.49	0.98; 2.27		93	19	56	130	0.20	68	15	16	7	84	17	10	6
Potowari	304	0.6%	185	248	3.01	1.60; 5.64	3.53	2.16; 5.79		182	80	26	338	0.44	117	69	63	59	180	80	3	3
Punjabi	10,452	20.8%	94	111	1.37	1.12; 1.68	1.58	1.29; 1.93		92	5	83	102	0.05	56	4	23	2	79	4	15	2
Pushto	11,257	22.4%	70	70	1.00		1.00			70	5	61	79	0.07	41	4	17	2	58	4	12	2
Shina	2,826	5.6%	92	91	1.35	0.86; 2.13	1.30	0.84; 2.02		92	11	72	113	0.11	38	7	37	6	75	10	19	4
Sindhi	4,938	9.8%	109	107	1.57	1.26; 1.97	1.53	1.22; 1.93		108	7	94	123	0.07	62	7	26	4	88	7	22	4
Siraiki	5,100	10.2%	120	121	1.77	1.43; 2.19	1.72	1.38; 2.14		119	9	102	137	0.07	67	8	29	4	96	9	25	4
Urdu	3,949	7.9%	75	120	1.12	0.84; 1.49	1.72	1.28; 2.29		74	9	57	92	0.12	46	7	19	4	65	8	10	3
Paraguay (2016 - MICS) [b]									8.5													
Speaks another language	461	3.2%	13	17	0.53	0.11; 2.55	0.71	0.11; 4.69	(2.4; 14.5)	13	11	0	35	0.87	13	11	0	0	13	11	0	0
Speaks Guarani and Spanish	3,817	26.6%	16	18	0.67	0.33; 1.34	0.74	0.36; 1.52		16	4	7	25	0.28	5	2	10	4	15	4	1	1
Speaks only Guarani	6,702	46.7%	24	24	1.00		1.00			25	4	18	33	0.15	12	3	10	3	22	4	3	1
Speaks only Spanish	2,261	15.8%	8	11	0.34	0.15; 0.78	0.46	0.19; 1.14		8	2	4	13	0.29	6	2	3	1	8	2	0	0
Indigenous	1,114	7.8%	71	56	3.04	1.82; 5.07	2.31	1.02; 5.21		74	18	38	109	0.25	11	6	51	15	62	17	13	6
Philippines (2013 - DHS) [b]									6.6													

Crude and adjusted under-five mortality rates and respective rate ratios by ethnic groups, estimated using the R procedure.

Mortality rates and standard errors by age and ethnic groups, estimated by DHS-*syncmrates* procedure.

Ethnicity	N	Share (%)	Crude U5MR	Adjusted U5MR	Crude analyses		Adjusted analyses [c]		Crude ratio between highest and lowest U5MR groups (95% CI)	Under-five					Neonatal		Post-neonatal		Infant		Child	
					U5MR ratio	95% CI	U5MR ratio	95% CI		Rate	s.e.	LL	UL	CV	Rate	s.e.	Rate	s.e.	Rate	s.e.	Rate	s.e.
Bicolano	1,883	5.9%	27	33	0.62	0.38; 1.00	0.73	0.45; 1.18	(2.7; 10.4)	26	6	15	37	0.22	13	4	2	2	15	4	11	4
Cebuano	6,409	20.2%	32	36	0.70	0.52; 0.95	0.80	0.59; 1.08		32	3	26	37	0.09	17	2	8	1	24	3	7	2
Ilocano	2,595	8.2%	35	43	0.74	0.49; 1.13	0.96	0.63; 1.47		35	7	22	48	0.20	17	4	10	3	27	6	8	3
Ilonggo	2,788	8.8%	28	33	0.62	0.42; 0.92	0.73	0.50; 1.06		28	4	20	36	0.15	14	3	9	3	23	4	5	2
Kapampangan	498	1.6%	13	18	0.28	0.10; 0.84	0.41	0.15; 1.15		13	7	0	27	0.54	5	5	0	0	5	5	8	4
Maranso	1,119	3.5%	84	84	2.04	1.18; 3.51	1.88	1.11; 3.18		85	12	61	109	0.14	11	6	23	7	34	8	53	10
**Other**	7,276	23.0%	45	45	1.00		1.00			45	4	38	52	0.08	16	2	17	2	33	3	13	2
Tagalog	7,135	22.5%	23	34	0.53	0.38; 0.73	0.77	0.55; 1.07		23	3	18	28	0.11	11	2	7	2	18	2	5	1
Tausog	836	2.6%	51	46	1.05	0.65; 1.70	1.03	0.66; 1.61		52	12	29	75	0.23	10	5	30	9	39	10	13	4
Waray	1,129	3.6%	29	34	0.66	0.38; 1.16	0.75	0.43; 1.30		29	8	15	44	0.26	8	4	9	5	17	6	12	4
Senegal (2016 - DHS) [a]									1.5													
Diola	608	2.7%	64	121	1.14	0.49; 2.64	1.70	0.69; 4.18	(0.9; 2.0)	63	21	22	104	0.33	42	19	12	8	54	21	9	5
Foreigner	549	2.4%	71	96	1.04	0.71; 1.53	1.35	0.93; 1.95		64	13	38	89	0.21	25	8	24	8	49	11	15	7
Mandingue	2,260	9.9%	68	73	0.88	0.64; 1.19	1.02	0.76; 1.37		69	9	51	86	0.13	21	4	23	9	44	9	26	5
Other	870	3.8%	56	76	0.79	0.48; 1.29	1.06	0.64; 1.77		56	17	22	89	0.31	23	14	18	8	42	17	15	3
**Poular**	8,149	35.8%	71	71	1.00		1.00			72	5	62	82	0.07	25	2	17	2	42	3	31	4
Serer	2,938	12.9%	57	71	0.84	0.58; 1.22	1.00	0.70; 1.42		57	7	43	71	0.13	25	6	17	4	42	6	15	4
Wolof	7,172	31.5%	48	63	0.70	0.52; 0.93	0.88	0.67; 1.15		49	4	41	56	0.08	22	3	13	2	35	4	14	2
Sierra Leone (2013 - DHS) [a]									1.8													
Fullah	1,410	3.0%	113	114	0.57	0.40; 0.80	0.59	0.42; 0.84	(1.3; 2.2)	117	14	89	145	0.12	38	9	43	9	81	11	39	10
Kono	2,441	5.2%	160	159	0.84	0.68; 1.03	0.83	0.68; 1.01		167	12	143	190	0.07	40	7	61	9	101	10	73	10
Koranko	1,939	4.1%	198	190	1.02	0.86; 1.20	0.99	0.84; 1.18		206	15	176	236	0.07	48	7	63	9	111	12	106	12
Limba	2,994	6.3%	133	131	0.67	0.53; 0.85	0.68	0.54; 0.87		137	10	118	156	0.07	48	9	46	5	95	9	47	6
Loko	1,086	2.3%	142	140	0.73	0.55; 0.97	0.73	0.55; 0.97		141	18	106	175	0.12	57	12	37	8	94	15	51	9
Mandingo	1,154	2.4%	164	175	0.85	0.65; 1.12	0.91	0.70; 1.20		167	21	127	208	0.12	74	17	53	11	128	21	46	10
**Mende**	17,003	35.9%	192	192	1.00		1.00			194	6	184	205	0.03	45	2	82	3	127	4	77	4
Other	2,454	5.2%	153	152	0.80	0.63; 1.01	0.79	0.62; 1.01		162	14	135	189	0.08	45	7	47	8	93	10	76	10
Sherbro	1,220	2.6%	179	165	0.87	0.68; 1.11	0.86	0.67; 1.10		177	16	145	208	0.09	31	7	74	12	104	15	81	15
Temne	15,240	32.2%	168	163	0.85	0.75; 0.96	0.85	0.75; 0.96		169	5	160	179	0.03	37	2	64	4	102	4	76	3
South Africa (2016 - DHS) [a]									1.8													
**Black / African**	12,361	87.4%	52	52	1.00		1.00		(1.0; 2.7)	52	4	45	60	0.07	25	3	20	2	45	4	8	1
Coloured	1,394	9.9%	40	45	0.75	0.25; 2.23	0.86	0.29; 2.55		39	11	17	62	0.29	27	9	6	4	33	11	7	4
White	276	2.0%	29	31	0.53	0.19; 1.45	0.58	0.18; 1.89		28	13	3	54	0.46	18	11	5	5	23	12	5	5
Togo (2013 - DHS) [a]									2.6													
Adja-ewe/mina	6,977	26.6%	75	88	0.66	0.54; 0.80	0.80	0.64; 0.99	(1.7; 3.5)	77	5	67	87	0.07	27	3	18	3	45	4	33	9
Akposso/akebou	1,030	3.9%	102	119	0.93	0.66; 1.30	1.08	0.75; 1.56		106	12	82	131	0.12	51	8	21	6	72	9	37	10
Ana-ife	706	2.7%	91	98	0.81	0.55; 1.22	0.89	0.59; 1.35		88	17	55	121	0.19	41	12	16	9	58	15	32	4
Foreigner	1,009	3.8%	46	61	0.40	0.26; 0.63	0.56	0.35; 0.88		47	10	27	66	0.21	19	7	15	6	34	8	13	4
Kabye/tem	7,151	27.2%	103	116	0.94	0.79; 1.12	1.06	0.87; 1.28		106	7	93	119	0.06	30	3	29	3	59	4	50	15

Crude and adjusted under-five mortality rates and respective rate ratios by ethnic groups, estimated using the R procedure.

Mortality rates and standard errors by age and ethnic groups, estimated by DHS-*syncmrates* procedure.

Ethnicity	N	Share (%)	Crude U5MR	Adjusted U5MR	Crude analyses		Adjusted analyses [c]		Crude ratio between highest and lowest U5MR groups (95% CI)	Under-five					Neonatal		Post-neonatal		Infant		Child	
					U5MR ratio	95% CI	U5MR ratio	95% CI		Rate	s.e.	LL	UL	CV	Rate	s.e.	Rate	s.e.	Rate	s.e.	Rate	s.e.
Other	827	3.1%	121	120	1.10	0.76; 1.59	1.09	0.76; 1.56		124	16	93	156	0.13	24	7	38	7	62	11	66	4
Para-gourma/akan	8,452	32.2%	110	110	1.00		1.00			113	5	103	124	0.05	29	2	29	2	57	3	59	3
Uganda (2016 - DHS) [a]									3.3													
Acholi	3,476	6.0%	69	50	1.36	1.00; 1.84	0.95	0.69; 1.30	(2.2; 4.4)	71	6	58	83	0.09	32	4	16	3	48	5	23	4
Alur	1,662	2.9%	106	72	1.96	1.43; 2.69	1.35	0.99; 1.84		108	12	84	131	0.11	37	7	31	7	68	9	42	7
Aringa	644	1.1%	67	46	1.25	0.59; 2.66	0.86	0.41; 1.81		69	13	43	95	0.19	25	10	11	5	36	12	34	9
Bafumbira	1,076	1.9%	82	62	1.58	0.89; 2.78	1.16	0.67; 2.03		80	13	55	105	0.16	25	7	20	7	45	9	37	11
Baganda	6,514	11.2%	53	53	1.00		1.00			54	4	46	63	0.08	27	3	11	2	38	4	18	3
Bagisu	3,206	5.5%	68	55	1.26	0.94; 1.70	1.04	0.76; 1.41		69	7	56	82	0.10	20	4	17	3	38	6	32	5
Bagwere	1,193	2.1%	73	57	1.40	0.94; 2.07	1.08	0.73; 1.59		73	11	51	94	0.15	31	8	15	5	47	9	28	7
Bakiga	4,523	7.8%	88	71	1.72	1.31; 2.26	1.33	1.02; 1.73		90	7	76	104	0.08	30	4	24	3	55	5	37	5
Bakonzo	1,260	2.2%	56	47	1.07	0.76; 1.49	0.89	0.65; 1.21		55	10	36	73	0.17	28	6	11	5	39	8	16	5
Banyankore	4,940	8.5%	69	58	1.30	0.98; 1.74	1.08	0.82; 1.44		71	5	61	81	0.07	24	3	20	3	44	4	27	4
Banyarwanda	1,116	1.9%	79	58	1.38	0.94; 2.03	1.08	0.74; 1.59		83	14	56	109	0.16	35	8	20	8	55	9	30	11
Banyole	1,101	1.9%	76	57	1.41	1.01; 1.99	1.08	0.77; 1.51		80	11	59	101	0.13	14	6	30	9	44	11	38	9
Banyoro	1,640	2.8%	85	76	1.64	1.19; 2.25	1.44	1.05; 1.97		82	10	62	102	0.12	34	6	24	5	58	7	26	8
Baruli	302	0.5%	73	62	1.42	0.47; 4.27	1.16	0.39; 3.46		77	22	35	120	0.28	28	14	25	14	52	18	26	14
Basamia	1,024	1.8%	111	91	2.14	1.58; 2.91	1.72	1.27; 2.33		111	16	79	142	0.15	33	10	48	11	81	13	32	10
Basoga	3,965	6.8%	84	71	1.57	1.19; 2.06	1.33	1.02; 1.74		84	5	73	94	0.06	35	4	22	4	57	4	28	5
Batoro	1,450	2.5%	73	59	1.33	0.91; 1.94	1.11	0.76; 1.62		72	8	56	88	0.11	22	5	21	5	44	7	30	5
Dodoth	431	0.7%	83	44	1.57	1.02; 2.41	0.83	0.53; 1.29		83	19	45	121	0.23	20	9	38	13	58	16	27	10
Ethur	271	0.5%	56	33	0.92	0.19; 4.56	0.61	0.12; 3.15		55	16	23	87	0.30	37	14	12	8	49	17	6	5
Iteso	5,282	9.1%	57	44	1.09	0.82; 1.45	0.82	0.61; 1.10		59	5	48	69	0.09	22	3	20	3	42	4	18	3
Jie	414	0.7%	144	79	2.91	2.00; 4.22	1.49	0.98; 2.26		142	31	82	203	0.22	61	22	61	16	121	27	24	11
Jopadhola	1,061	1.8%	62	52	1.26	0.78; 2.03	0.99	0.61; 1.58		61	10	42	79	0.16	15	5	17	5	32	6	29	8
Kakwa	206	0.4%	106	76	2.08	0.96; 4.50	1.44	0.62; 3.31		103	29	46	161	0.28	85	27	18	11	103	29	0	0
Karimojong	982	1.7%	86	49	1.65	1.18; 2.31	0.91	0.63; 1.31		85	15	55	115	0.18	11	5	42	11	53	12	34	8
Kumam	512	0.9%	44	31	0.83	0.41; 1.68	0.58	0.29; 1.17		45	14	17	73	0.32	21	9	13	7	34	12	11	7
Lango	4,161	7.2%	63	46	1.20	0.88; 1.63	0.87	0.65; 1.18		64	5	54	74	0.08	28	4	15	3	43	5	22	3
Lugbara	1,746	3.0%	87	65	1.68	1.18; 2.40	1.23	0.87; 1.73		88	10	68	108	0.12	27	5	27	6	54	7	36	7
Madi	555	1.0%	62	42	1.10	0.76; 1.60	0.79	0.53; 1.17		61	15	32	91	0.25	8	5	24	10	31	11	31	12
Other	1,273	2.2%	94	71	1.79	1.20; 2.67	1.33	0.91; 1.93		96	15	67	124	0.15	26	8	32	11	58	12	40	9
Pokot	275	0.5%	70	37	1.31	0.91; 1.88	0.70	0.47; 1.02		72	18	36	107	0.25	17	10	28	13	45	14	28	11
Sabiny	357	0.6%	58	47	1.15	0.62; 2.12	0.88	0.48; 1.62		58	20	19	97	0.34	22	8	31	15	54	19	5	4
Zambia (2013 - DHS) [a]									8.2													
African	415	0.8%	117	109	1.27	0.76; 2.12	1.31	0.81; 2.13	(4.9; 11.4)	119	28	64	174	0.24	42	19	36	19	78	29	44	16
Bemba	11,499	23.4%	83	83	1.00		1.00			84	3	78	91	0.04	23	2	25	3	48	3	38	3
Bisa	704	1.4%	84	77	1.05	0.68; 1.64	0.93	0.59; 1.46		89	16	58	119	0.17	30	10	30	9	60	13	30	10
Bwile	242	0.5%	220	196	2.74	1.83; 4.11	2.36	1.59; 3.51		215	40	137	294	0.19	48	17	93	27	141	31	86	28

Crude and adjusted under-five mortality rates and respective rate ratios by ethnic groups, estimated using the R procedure.

Ethnicity	N	Share (%)	Crude U5MR	Adjusted U5MR	Crude analyses		Adjusted analyses [c]		Crude ratio between highest and lowest U5MR groups (95% CI)
					U5MR ratio	95% CI	U5MR ratio	95% CI	
Chewa	3,335	6.8%	88	87	1.10	0.88; 1.38	1.05	0.83; 1.31	
Chokwe	312	0.6%	28	27	0.32	0.14; 0.69	0.32	0.15; 0.70	
Ila	231	0.5%	55	72	0.80	0.37; 1.74	0.87	0.42; 1.81	
Kaonde	1,899	3.9%	65	67	0.80	0.56; 1.13	0.81	0.57; 1.16	
Kunda	262	0.5%	27	28	0.31	0.15; 0.66	0.34	0.16; 0.73	
Lala	1,589	3.2%	118	107	1.39	1.02; 1.90	1.29	0.95; 1.74	
Lamba	1,162	2.4%	84	84	1.01	0.71; 1.45	1.01	0.71; 1.44	
Lenje	674	1.4%	58	51	0.61	0.35; 1.07	0.62	0.35; 1.10	
Lozi	2,867	5.8%	81	82	1.01	0.69; 1.47	1.00	0.69; 1.43	
Luchazi	223	0.5%	68	53	0.71	0.17; 2.92	0.64	0.16; 2.62	
Lunda (luapula)	291	0.6%	62	68	0.78	0.38; 1.58	0.82	0.41; 1.65	
Lunda (north-western)	1,863	3.8%	61	57	0.73	0.53; 1.02	0.69	0.50; 0.95	
Lungu	427	0.9%	68	63	0.79	0.45; 1.40	0.76	0.42; 1.35	
Luvale	1,443	2.9%	46	42	0.53	0.34; 0.83	0.51	0.32; 0.80	
Mambwe	1,589	3.2%	69	74	0.90	0.61; 1.34	0.89	0.61; 1.30	
Mbunda	809	1.6%	61	48	0.65	0.43; 0.98	0.58	0.39; 0.87	
Namwanga	1,650	3.4%	72	72	0.85	0.64; 1.14	0.87	0.66; 1.16	
Ngoni	1,639	3.3%	105	101	1.20	0.88; 1.62	1.22	0.90; 1.64	
Ngumbo	287	0.6%	31	20	0.27	0.14; 0.50	0.24	0.13; 0.44	
Nsenga	2,131	4.3%	89	90	1.10	0.80; 1.50	1.09	0.81; 1.46	
Other language	596	1.2%	71	65	0.89	0.54; 1.45	0.78	0.47; 1.30	
Senga	447	0.9%	108	109	1.42	0.94; 2.15	1.32	0.90; 1.94	
Soli	260	0.5%	88	96	1.08	0.5; 2.34	1.16	0.52; 2.56	
Tabwa	262	0.5%	124	113	1.57	0.91; 2.70	1.37	0.81; 2.31	
Toka-leya	213	0.4%	65	75	0.93	0.36; 2.41	0.91	0.35; 2.36	
Tonga	5,431	11.0%	62	63	0.74	0.61; 0.91	0.76	0.62; 0.94	
Tumbuka	2,097	4.3%	107	108	1.31	1.04; 1.65	1.30	1.03; 1.64	
Ushi	1,138	2.3%	58	58	0.74	0.42; 1.32	0.70	0.39; 1.26	

Mortality rates and standard errors by age and ethnic groups, estimated by DHS-*syncmrates* procedure.

Ethnicity	Under-five					Neonatal		Post-neonatal		Infant		Child	
	Rate	s.e.	LL	UL	CV	Rate	s.e.	Rate	s.e.	Rate	s.e.	Rate	s.e.
Chewa	87	7	74	101	0.08	25	4	27	5	52	6	38	5
Chokwe	27	14	0	54	0.51	0	0	13	9	13	9	15	12
Ila	61	17	28	94	0.28	27	12	26	11	53	16	9	6
Kaonde	65	8	49	81	0.13	23	6	22	5	45	7	21	4
Kunda	27	11	5	48	0.41	22	11	0	0	22	11	5	6
Lala	118	13	93	143	0.11	26	7	32	7	59	10	63	9
Lamba	81	12	58	104	0.15	40	8	15	5	54	10	28	7
Lenje	56	14	30	83	0.24	34	11	8	4	43	11	14	7
Lozi	82	10	64	101	0.12	27	5	20	5	47	7	37	7
Luchazi	68	33	4	132	0.48	7	4	7	8	13	10	55	30
Lunda (luapula)	57	26	7	107	0.45	27	19	8	8	35	21	22	15
Lunda (north-western)	62	9	44	80	0.15	28	6	12	5	40	7	23	5
Lungu	70	16	39	101	0.23	8	4	25	8	32	9	39	14
Luvale	46	10	27	65	0.21	11	4	15	5	26	7	20	7
Mambwe	73	11	51	95	0.16	22	6	25	7	47	9	27	6
Mbunda	60	10	40	79	0.17	28	7	12	5	40	9	21	6
Namwanga	72	9	54	90	0.13	19	5	25	6	44	8	29	7
Ngoni	107	13	82	132	0.12	37	7	27	7	63	10	46	9
Ngumbo	42	18	6	78	0.44	7	7	0	0	7	7	35	18
Nsenga	89	11	68	110	0.12	21	5	32	6	53	8	38	6
Other language	71	14	43	98	0.20	22	8	27	9	49	13	23	11
Senga	103	21	61	145	0.21	32	12	38	14	71	17	35	14
Soli	88	41	7	169	0.47	39	26	24	19	62	34	27	15
Tabwa	127	30	67	187	0.24	45	18	54	23	99	28	31	17
Toka-leya	66	32	3	128	0.49	15	14	30	16	45	22	22	21
Tonga	62	5	52	72	0.08	19	3	17	3	37	4	26	4
Tumbuka	108	9	89	126	0.09	44	7	27	5	70	8	40	7
Ushi	58	11	37	79	0.18	20	6	15	6	36	8	23	7

[a] Groups according to ethnicity.

[b] Groups according to language.

[c] Adjusted for area of residence, wealth index, and education. Reference groups in each country are highlighted in bold font.

Annex B. Estimation of mortality rates. Rates refer to the 10-year period preceding each survey.

The traditional approach used in DHS and MICS to estimate under-five mortality based upon birth histories consists in using a synthetic cohort life table approach in which mortality probabilities are first computed for small age segments (based on a real cohort experience in these age segments) (https://dhsprogram.com/pubs/pdf/DHSG1/Guide_to_DHS_Statistics_29Oct2012_DHSG1.pdf). Because there are incomplete cohorts at the beginning and end of the period for which rates are computed, DHS has a set of rules to account for partial exposure. For example, the routine retains half of the deaths in the cohorts that are only partially exposed across two age segments, and a similar correction is undertaken for the denominators. Once the age-specific mortality rates have been obtained, they are combined to estimate under-five mortality. The *syncmrates* procedure in Stata adopts the DHS approach and produces mortality rates that are exactly the same as in DHS. (Edoardo Masset, 2016. "**SYNCMRATES: Stata module to compute child mortality rates using synthetic cohort probabilities**," Statistical Software Components S458149, Boston College Department of Economics.)

However, this approach is difficult to translate into a regression framework, for example, when one wishes to adjust for covariates. One can avoid this limitation by computing *m-rates* (deaths over person-years) using survival analysis. This has the extra advantage of allowing the use of Poisson regression for covariate adjustment and was adopted in the present set of analyses.

The survival analysis procedure starts by computing *m-rates*, which are then converted into *q-rates* (probabilities), also in narrow age segments. The only parameter needed to convert m-rates into q-rates is the average time spent in the interval by those who died in the interval, which is known as ${}_n a_x$. This parameter is assumed to be the same across all ethnic groups, and to correspond to half the duration of the time intervals, which consist of months below age one year, and years from ages 1-5 years. Therefore, this method is very close to the DHS approach. The differences lie in the extra step of firstly computing death rates (deaths over person-years) and converting them into probabilities. Because mortality is estimated for narrow age groups, the results are not very sensitive to the ${}_n a_x$. parameter, and as a result, both the overall under-five mortality rates and the rate ratios are very similar to those published in DHS reports (and calculated using *syncmrates* in Stata), for example when comparing the national average or the urban/rural rate ratios.

We calculated under-five mortality by ethnic group using both the DHS-*syncmrates* procedure and the survival analysis procedures for 36 countries. Supplementary figure 1 shows that there was very close agreement between the two methods in the estimates of under-five mortality rates.

Supplementary Figure 1. Comparison of DHS-*syncmrates* and survival analysis estimates of under-five mortality rate ratios by ethnic group in 36 countries. Each dot represents one ethnic group, and the largest group in each country is the reference (rate ratio of 1.0).

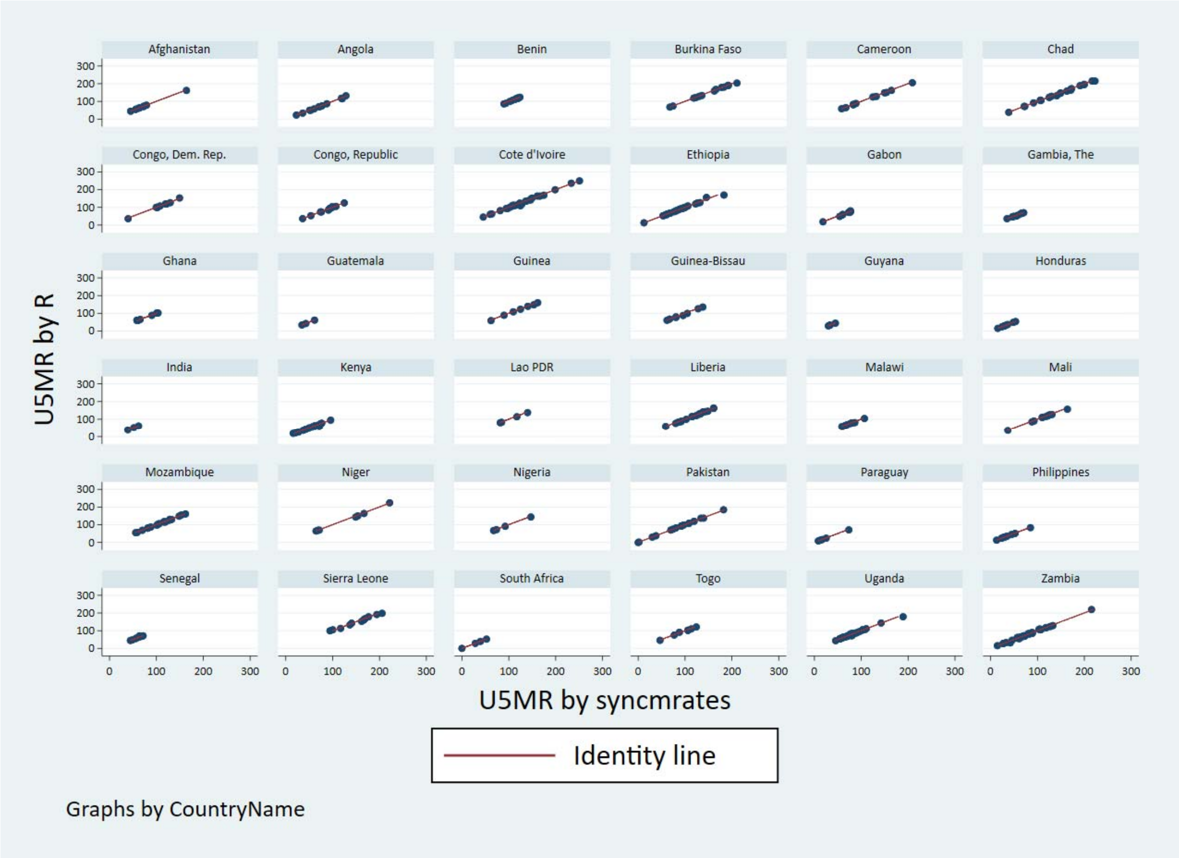

REFERENCES:

Masset E. SYNCMRATES: Stata module to compute child mortality rates using synthetic cohort probabilities. (Statistical Software Components S458149). Boston: Boston College Department of Economics.; 2016.

Eaton J. Analysis of demographic indicators from Demographic and Health Surveys (DHS) and other household surveys. 2019. https://github.com/mrc-ide/demogsurv (accessed 9 August 2019.

Annex C. Summary measures of inequality

Two summary measures were calculated for ethnic group inequality within each country. Their respective formulae are shown below.

The magnitude of relative inequalities was estimated by Theil's index,[1] which takes into account the proportion of the population in each group and the mortality ratios in each ethnicity relative to the national mean value. A value of zero shows that there is no inequality, and the value increases with the degree of inequality, with no upper bound. The index is most influenced by large ethnic groups with mortality rates that are substantially different from the national rate. Index values may be scaled to facilitate interpretation. In our analyses the original values were multiplied by 1,000.

For absolute inequalities, we calculated between-group variance, which starts at zero when there is no inequality and takes positive values as inequalities increase, based on the size of each ethnic group and the difference from the overall mean mortality rate.

We estimated the standard errors for both indices using bootstrapping procedures that involved resampling 50 times with replacement for each ethnicity in each country. When the lower 95% confidence limit for the index was below zero, indicating no statistical evidence of inequality, we truncated it at zero as negative values are not interpretable.

Theil's index formula

$$Theil = \frac{1}{N} * \sum_{i=1}^{N} \left(\frac{x_i}{\bar{x}} * \ln \frac{x_i}{\bar{x}} \right) * 1000$$

where

N – total number of ethnic groups in a country

x_i – under-five mortality rate of the i_{th} ethnic group of a country

$\bar{x}$ – mean under-five mortality rate between all ethnic groups of a country

Between-group variance (BGV) formula

$$BGV = \frac{1}{N} * \sum_{i=1}^{N} n_i * (x_i - \bar{x})^2$$

where

N – total number of ethnic groups in a country

n_i – number of individuals from the i_{th} ethnic group of a country

x_i – under-five mortality rate of the i_{th} ethnic group of a country

$\bar{x}$ – mean under-five mortality rate between all ethnic groups of a country

The table below shows the values of these indices for each country.

Interpretation of the summary indices

The equity literature recommends presentation of both absolute and relative inequality measures, as these may provide contrasting (and apparently contradictory) results.[1-3]

For example, Theil's index much higher in the Philippines (61.5) with U5MR ranging from 13 to 85, than in Mozambique (26.8) with U5MR ranging from 56 to 162. This is because relative inequality is much greater in the Philippines (85 over 13 for the extreme groups ratio) than in Mozambique (162 over 56). In contrast, BGV – which reflects the difference between groups - is wider in Mozambique (BGV = 589) than in the Philippines (BGV = 169), as would be expected given that absolute inequality is larger in Mozambique.

An additional issue that affects interpretation of both indices is that more populous ethnic groups receive greater weight in the formula than smaller groups. For example, BGV values in Angola, Guinea Bissau, and Pakistan are equal to 511.4, 513.2 and 564.6 respectively. In Angola, the difference between the highest and lowest U5MR by ethnic group is equal to 109, whereas in Guinea-Bissau it equals 58 and in Pakistan 155. Taken at face value, this would suggest that absolute inequalities in Pakistan would be wider than in the Angola or Guinea-Bissau. However, the BGV formula (as well as the formula for Theil's index) give greater emphasis to the largest groups in the sample. In Pakistan, the two largest groups are the Pashtu (U5MR=70) and Punjabi (94); in Angola, the Portuguese (70) and Umbundu (118), and in Guinea-Bissau the Crioulo (80) and Fula (136). Therefore, differences between the two largest groups tend to be less marked than differences among the extreme groups, some of which are relatively small.

These examples show that the interpretation of both summary indices is complex because it depends not only on the metric (absolute or relative inequality) but also on the size and U5MR of the different groups. This problem affects all summary indices of inequality among unordered groups, such as ethnicity or geographical regions in a country. Nevertheless, these indices are widely used to summarize the degree of inequality taking the whole population into account, and approach that complements the simpler comparison between the highest and lowest mortality groups.

Country	Year	Type of survey	Variable	Total number of births	Ethnic groups	Theil's index for inequality 95%CI¹		Between-group variance 95%CI	
Afghanistan	2015	DHS	Ethnicity	125,488	9	39.9	30.9;48.9	459.8	344.6;574.9
Angola	2015	DHS	Language	41,999	12	40.1	28.1;52.0	511.4	364.4;658.4
Benin	2014	MICS	Ethnicity	45,183	10	2.4	0.0;5.2	58.8	0.0;120.4
Burkina Faso	2010	DHS	Ethnicity	55,853	12	19.7	13.6;25.8	863.4	592.0;1134.8
Cameroon	2014	MICS	Ethnicity	26,040	11	68.0	50.7;85.4	1,579.6	1175.7;1983.6
Chad	2014	DHS	Ethnicity	67,356	22	50.4	43.1;57.7	1,831.4	1532.2;2130.6
Congo, Dem. Rep.	2013	DHS	Ethnicity	59,074	9	5.6	2.5;8.7	134.7	53.2;216.2
Congo, Republic	2011	DHS	Ethnicity	31,688	11	10.0	2.8;17.2	145.2	19.3;271.0
Côte d'Ivoire	2011	DHS	Ethnicity	26,317	23	8.3	26.1;57.0	45.8	771.6;1932.6
Ethiopia	2016	DHS	Ethnicity	39,494	19	41.6	15.1;38.8	1,352.1	176.5;572.9
Gabon	2012	DHS	Ethnicity	20,619	9	26.9	4.9;33.8	374.7	0.0;294.8
Gambia, The	2013	DHS	Ethnicity	26,179	10	19.3	0.0;16.6	138.9	0.0;122.0
Ghana	2014	DHS	Ethnicity	23,117	9	7.1	10.1;39.4	51.4	94.1;460.2
Guatemala	2014	DHS	Ethnicity	55,300	3	24.8	0.0;14.5	277.1	0.0;46.2
Guinea	2012	DHS	Ethnicity	27,683	7	7.2	7.9;21.0	22.1	282.2;744.3
Guinea-Bissau	2014	MICS	Language	27,477	7	14.5	23.7;49.4	513.2	490.3;1008.1
Guyana	2014	MICS	Ethnicity	11,122	4	36.5	0.0;36.1	749.2	0.0;78.7
Honduras	2011	DHS	Ethnicity	48,893	7	13.8	0.0;26.5	37.1	0.0;68.6
India	2015	DHS	Ethnicity	1,265,049	5	12.5	7.1;9.6	27.3	39.0;52.5
Kenya	2014	DHS	Ethnicity	83,571	23	59.3	45.2;73.4	364.8	266.0;463.7
Lao PDR	2011	MICS	Ethnicity	56,710	4	30.3	22.9;37.8	704.6	540.7;868.5
Liberia	2013	DHS	Language	30,713	18	15.2	8.3;22.0	400.1	199.0;601.2
Malawi	2015	DHS	Ethnicity	68,074	11	7.0	3.2;10.8	77.8	35.6;120.1

Mali	2015	MICS	Ethnicity	55,772	11	31.0	24.4;37.5	648.8	515.9;781.7
Mozambique	2011	DHS	Ethnicity	37,877	19	26.8	17.7;35.8	589.9	380.2;799.7
Niger	2012	DHS	Language	43,831	6	16.0	11.2;20.8	593.3	454.2;732.5
Nigeria	2016	MICS	Ethnicity	101,691	4	41.8	33.5;50.0	1,064.6	836.8;1292.4
Pakistan	2012	DHS	Language	49,913	15	31.7	21.0;42.3	564.6	388.6;740.6
Paraguay	2016	MICS	Language	14,355	5	174.5	94.4;254.5	249.0	144.8;353.2
Philippines	2013	DHS	Language	31,668	10	61.5	37.5;85.6	169.2	100.5;237.8
Senegal	2016	DHS	Ethnicity	22,546	7	13.6	0.0;27.5	99.6	0.0;222.4
Sierra Leone	2013	DHS	Ethnicity	46,941	10	7.1	4.2;10.0	419.4	262.7;576.0
South Africa	2016	DHS	Ethnicity	14,031	3	5.5	0.0;20.5	25.1	0.0;85.5
Togo	2013	DHS	Ethnicity	26,152	7	19.1	10.0;28.2	340.1	149.9;530.3
Uganda	2016	DHS	Ethnicity	56,618	31	22.0	13.2;30.8	247.9	146.0;349.9
Zambia	2013	DHS	Ethnicity	47,987	32	31.4	22.3;40.5	417.6	283.5;551.7
**Total**				**2,812,381**	**415**				

[1] Standard error estimated by resampling (with replacement) 50 times the observations of each ethnicity per country.

Annex D. Inequality measures for mortality by age group.

Country	Year	Type of survey	Theil's index of inequality					Between-group variance				
			U5MR	NMR	PNMR	IMR	CMR	U5MR	NMR	PNMR	IMR	CMR
Afghanistan	2015	DHS	39.9	10.2	70.5	30.8	108.5	459.8	14.9	137.0	217.8	65.2
Angola	2015	DHS	40.1	64.9	48.2	49.1	34.0	511.4	75.4	60.6	246.4	66.1
Benin	2014	MICS	2.4	21.8	12.4	8.6	8.1	58.8	51.4	25.0	74.6	41.3
Burkina Faso	2010	DHS	19.7	23.5	28.1	20.7	32.4	863.4	60.7	111.7	264.8	340.3
Cameroon	2014	MICS	68.0	90.4	57.5	60.5	108.2	1579.6	197.6	123.8	513.0	470.0
Chad	2014	DHS	50.4	36.3	69.1	46.5	72.4	1831.4	81.8	230.5	504.5	646.6
Congo, Dem. Rep.	2013	DHS	5.6	9.1	8.2	5.4	25.1	134.7	20.0	20.3	42.2	124.7
Congo, Republic	2011	DHS	10.0	33.9	39.9	22.9	13.3	145.2	44.1	51.5	118.5	37.7
Côte d'Ivoire	2011	DHS	41.6	84.4	50.9	49.7	75.1	1352.1	367.5	137.6	637.6	392.3
Ethiopia	2016	DHS	26.9	25.7	102.3	21.0	110.8	374.7	63.9	108.6	142.7	154.5
Gabon	2012	DHS	19.3	60.1	66.3	40.9	57.9	138.9	68.3	41.0	149.9	47.1
Gambia, The	2013	DHS	7.1	37.8	38.8	19.3	27.6	51.4	47.0	10.5	49.2	25.7
Ghana	2014	DHS	24.8	7.0	63.0	9.6	120.4	277.1	14.1	40.8	49.6	174.6
Guatemala	2014	DHS	7.2	4.9	10.7	6.4	30.3	22.1	3.1	4.7	13.4	4.5
Guinea	2012	DHS	14.5	13.7	41.7	16.0	20.4	513.2	43.0	104.4	184.1	158.7
Guinea-Bissau	2014	MICS	36.5	52.7	51.0	43.5	51.8	749.2	154.8	50.6	327.6	169.8
Guyana	2014	MICS	13.8	109.2	39.7	31.3	71.9	37.1	88.9	8.7	58.9	5.2
Honduras	2011	DHS	12.5	8.8	30.6	10.5	32.5	27.3	6.1	4.0	14.7	2.8
India	2015	DHS	8.3	5.0	9.3	6.0	28.4	45.8	9.1	2.5	21.0	6.9
Kenya	2014	DHS	59.3	32.1	135.7	27.9	260.1	364.8	35.1	85.9	84.0	141.4
Lao PDR	2011	MICS	30.3	36.0	22.1	23.7	115.2	704.6	130.2	92.1	370.7	98.7
Liberia	2013	DHS	15.2	54.4	27.6	22.9	34.6	400.1	137.5	78.8	234.4	140.8
Malawi	2015	DHS	7.0	15.8	26.4	8.4	18.5	77.8	25.6	22.5	37.2	30.3
Mali	2015	MICS	31.0	43.9	23.0	28.1	45.4	648.8	75.6	26.2	153.3	259.5
Mozambique	2011	DHS	26.8	47.8	52.2	27.6	54.2	589.9	90.2	154.6	266.1	170.0
Niger	2012	DHS	16.0	18.9	15.9	13.6	23.3	593.3	27.5	27.3	90.9	319.1
Nigeria	2016	MICS	41.8	5.5	32.8	14.0	120.4	1064.6	14.9	61.5	128.5	557.4
Pakistan	2012	DHS	31.7	35.0	67.9	28.8	96.0	564.6	197.8	85.4	360.1	60.8
Paraguay	2016	MICS	174.5	68.2	333.6	147.1	338.2	249.0	10.3	138.8	165.2	9.8
Philippines	2013	DHS	61.5	24.3	127.2	40.5	224.9	169.2	8.7	34.5	46.9	76.4
Senegal	2016	DHS	13.6	8.7	17.0	5.3	73.6	99.6	11.7	9.5	17.7	68.1
Sierra Leone	2013	DHS	7.1	12.1	20.9	8.3	16.6	419.4	48.8	181.3	200.9	165.8
South Africa	2016	DHS	5.5	1.1	40.3	6.3	2.2	25.1	1.3	20.0	20.8	0.3
Togo	2013	DHS	19.1	15.0	27.6	10.7	42.8	340.1	28.8	33.3	60.6	168.4
Uganda	2016	DHS	22.0	36.5	68.6	26.9	44.0	247.9	56.8	66.9	140.9	63.1
Zambia	2013	DHS	31.4	45.1	60.2	37.7	48.5	417.6	61.1	73.4	180.1	110.8
**Median**			**20.8**	**28.9**	**40.1**	**22.9**	**46.9**	**369.8**	**47.9**	**56.0**	**141.8**	**104.8**
**Mean**			**28.9**	**33.3**	**53.8**	**27.1**	**71.9**	**448.6**	**65.9**	**68.5**	**171.9**	**149.3**

Annex E. U5MR by ethnic groups in countries, according to regions of the world.

Eastern and Southern Africa

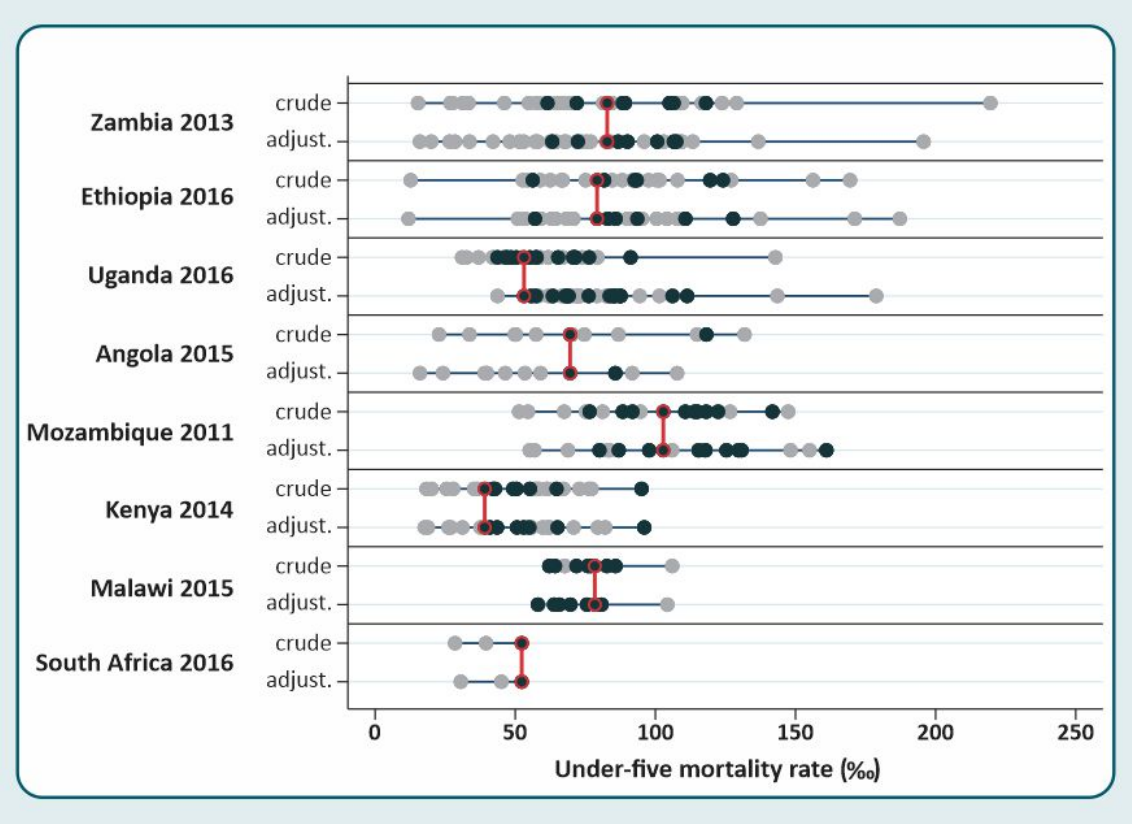

West and Central Africa

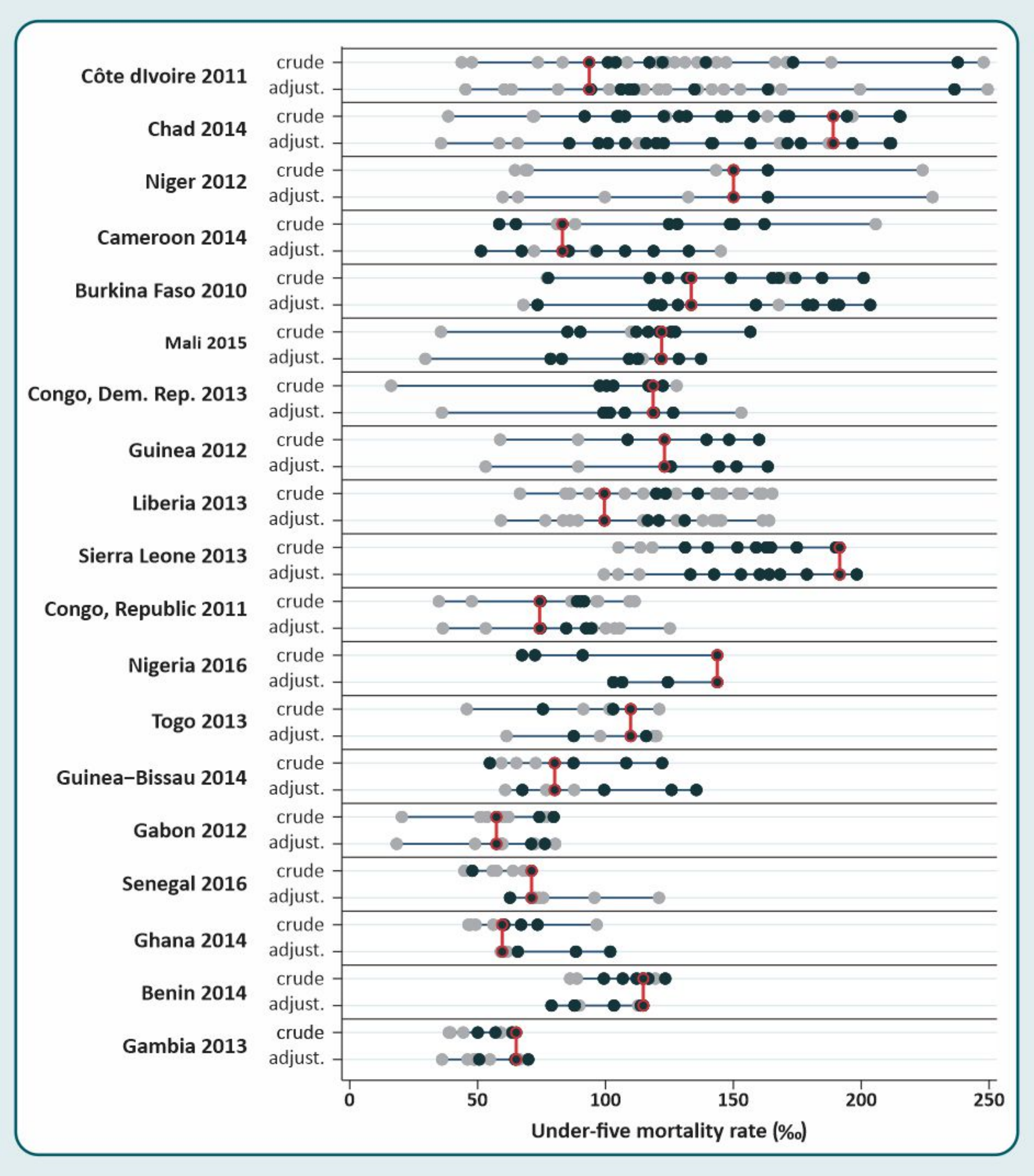

Countries from outside Africa

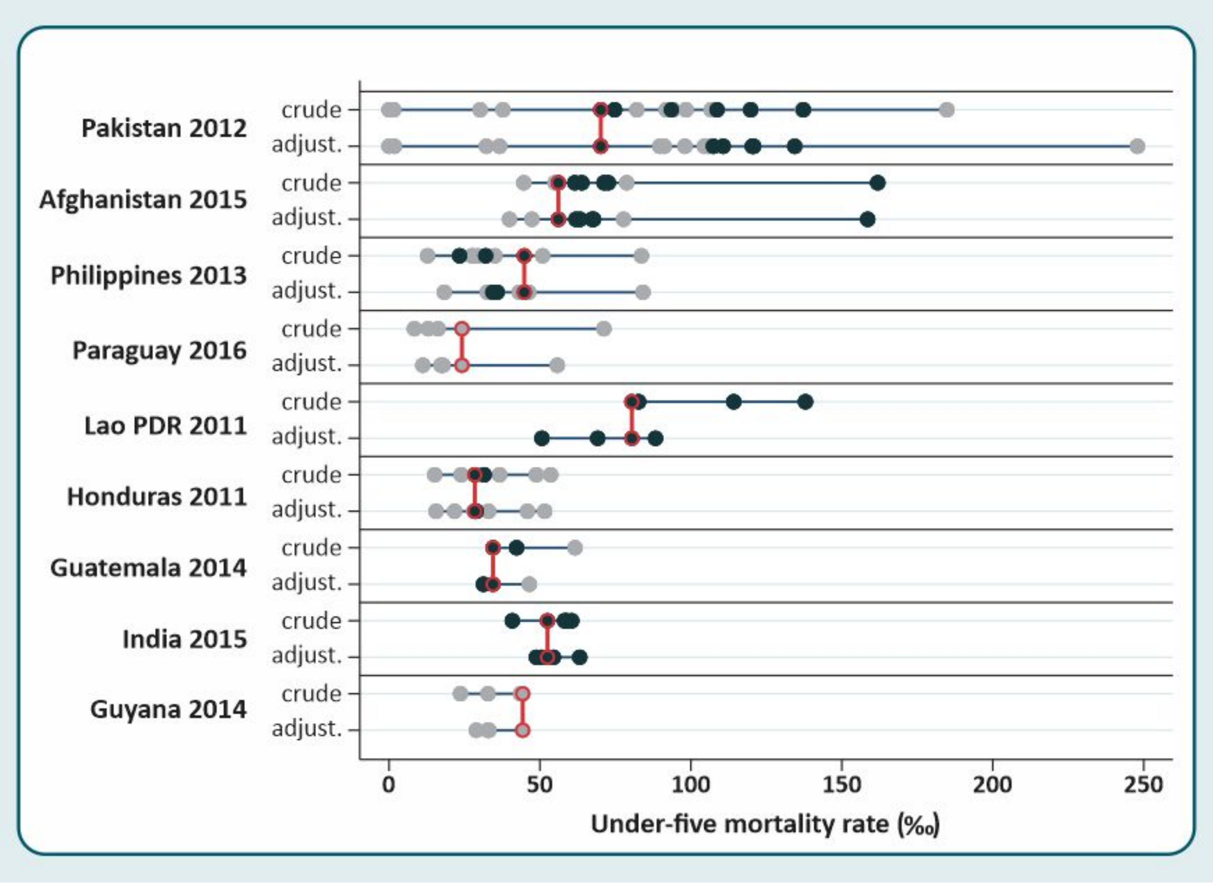

End of supplementary materials
